# Finding the right dose: a scoping review examining facilitation as an implementation strategy for evidence-based stroke care

**DOI:** 10.1186/s13012-025-01415-w

**Published:** 2025-01-13

**Authors:** Oyebola Fasugba, Heilok Cheng, Simeon Dale, Kelly Coughlan, Elizabeth McInnes, Dominique A. Cadilhac, Ngai W. Cheung, Kelvin Hill, Kirsty Page, Estela Sanjuan Menendez, Emily Neal, Vivien Pollnow, Julia Slark, Eileen Gilder, Anna Ranta, Christopher Levi, Jeremy M. Grimshaw, Sandy Middleton

**Affiliations:** 1 Nursing Research Institute, St Vincent’s Health Network Sydney, St Vincent’s Hospital Melbourne & Australian Catholic University, Level 5, deLacy Building, St. Vincent’s Hospital, 390 Victoria Street, Darlinghurst, 2010 New South Wales Australia; 2 School of Nursing, Midwifery and Paramedicine, Australian Catholic University, Sydney, Australia; 3 Stroke and Ageing Research, Department of Medicine, School of Clinical Sciences at Monash Health, Monash University, Victoria, Australia; 4 Stroke Theme, Florey Institute of Neuroscience and Mental Health, University of Melbourne, Heidelberg, Victoria Australia; 5 Centre for Diabetes and Endocrinology Research, Westmead Hospital and University of Sydney, New South Wales, Australia; 6 Stroke Foundation, Sydney, New South Wales Australia; 7 Vincent’s Health Network Sydney, New South Wales, Australia; 8 Vall d’Hebron Hospital Universitari Barcelona, Barcelona, Spain; 9 Prince of Wales Hospital, Randwick, New South Wales Australia; 10 School of Nursing, Faculty of Medical and Health Sciences, The University of Auckland, Auckland, New Zealand; 11 Department of Medicine, University of Otago Wellington, Wellington, New Zealand; 12 Department of Neurology, Wellington Hospital, Wellington, New Zealand; 13 John Hunter Hospital, Newcastle, New South Wales Australia; 14 Department of Medicine, University of Newcastle, New South Wales, Australia; 15 Department of Medicine, University of Ottawa, Ottawa, Ontario Canada; 16 Methodological and Implementation Research Program, Ottawa Hospital Research Institute, Ottawa, Canada

**Keywords:** Facilitation, Implementation Strategy, Dose, Intensity, Content, Scoping Review

## Abstract

**Background:**

Despite evidence supporting interventions that improve outcomes for patients with stroke, their implementation remains suboptimal. Facilitation can support implementation of research into clinical practice by helping people develop the strategies to implement change. However, variability in the amount (dose) and type of facilitation activities/facilitator roles that make up the facilitation strategies (content), may affect the effectiveness of facilitation. This review aimed to determine if, and how, facilitation dose is measured or reported and the type of facilitation strategies used to support adoption of stroke interventions in hospitals and subacute settings. We also assessed whether the included studies had reporting checklists or guidelines.

**Methods:**

The scoping review was based on Arksey and O’Malley’s framework. Cochrane, CINAHL and MEDLINE databases were searched to identify randomised trials and quasi-experimental studies of stroke interventions published between January 2017 and July 2023. Accompanying publications (quantitative, qualitative, mixed methods or process evaluation papers) from eligible studies were also included. Narrative data synthesis was undertaken.

**Results:**

Ten studies (23 papers) from 649 full-text papers met the inclusion criteria. Only two studies reported the total facilitation dose, measured as the frequency and duration of facilitation encounters. Authors of the remaining eight studies reported only the frequency and/or duration of varying facilitation activities but not the total dose. The facilitation activities included remote external facilitator support via ongoing telecommunication (phone calls, emails, teleconferences), continuous engagement from on-site internal facilitators, face-to-face workshops and/or education sessions from external or internal facilitators. Facilitator roles were broad: site-specific briefing, action planning and/or goal setting; identifying enablers and barriers to change; coaching, training, education or feedback; and network support. Only two studies included reporting checklists/guidelines to support researchers to describe interventions and implementation studies in sufficient detail to enable replication.

**Conclusions:**

There is a paucity of information on the measurement of facilitation dose and reporting on specific details of facilitation activities in stroke implementation studies. Detailed reporting of dose and content is needed to improve the scientific basis of facilitation as strategic support to enable improvements to stroke care. Development of a standardised measurement approach for facilitation dose would inform future research and translation of findings.

**Supplementary Information:**

The online version contains supplementary material available at 10.1186/s13012-025-01415-w.

Contributions to the literature • The facilitation dose and types of facilitation strategies (content) required for optimal intervention uptake is unknown.

• Despite reporting guidelines for intervention description and replication (TIDieR Guidelines) our review highlights a significant evidence gap regarding the measurement and reporting of facilitation dose and content in stroke.

• Findings illustrate the need for better reporting on specific details of intervention delivery to allow exploration of heterogeneity in the effects of facilitation.

• Implementation science researchers should develop and validate standardised methods (quantitative and qualitative) for describing facilitation dose and content, particularly external facilitation, to enable examination of facilitation impact on intervention effectiveness.

## Introduction

Achieving successful knowledge translation and implementation of evidence-based interventions in clinical practice is often difficult [1, 2]. In addition to the complexity and challenges, there remains a lack of knowledge about what strategies are most effective in changing clinician behaviour and successfully implementing evidence into practice [3].

Implementation frameworks highlight the need for appropriate facilitation to improve the potential of implementation success [3–5]. Facilitation refers to the process of providing help and support to individuals and teams to enable them achieve a specific goal [6]. It is ‘a process of interactive problem solving and support that occurs in a context of a recognized need for improvement and a supportive interpersonal relationship’ [7]. Facilitation may occur in various forms, using either an external facilitator, an internal facilitator, or a combination of both [6]. Facilitators are considered as ‘change agents’ or ‘champions’ and their key roles are to identify, engage, and connect stakeholders; facilitate collaboration including the development of implementation action plans; support communication and information sharing; and evaluate practice change [6, 8–10].

Despite evidence supporting interventions that improve outcomes for patients with stroke, implementation of these evidence-based stroke interventions remains suboptimal [11, 12]. Facilitation has the potential to improve stroke evidence translation and, thus, clinician practice. Facilitator roles that have been examined in published studies of stroke interventions and shown to be effective include external facilitators undertaking telephone contact and on‐site visits with clinicians to facilitate improvement in venous thromboembolism prevention for stroke patients [13]; internal clinical facilitators facilitating improvements in the organisation and delivery of stroke patient care [14]; and internal non‐clinical facilitators facilitating improvements in adherence to clinical processes of care [15].

Evidence in support of facilitation as an implementation strategy for increasing uptake of evidence-based interventions into clinical practice is mixed [13–16]. The specific reason for this is poorly understood due to a lack of conceptual clarity but may occur because of variability in the amount (dose) and types of facilitation activities/facilitator roles that make up the facilitation strategies (content) [17]. As a result, it is recommended that both the dose and content of facilitation are measured in effectiveness and comparative effectiveness studies [18]. This is particularly important to show the minimal dose and content required to obtain the strongest effect as well as to have a better understanding of the processes and mechanisms by which implementation strategies exert their effects [18, 19]. Despite its potential benefits, the facilitation dose and content required for optimal uptake of interventions, that is, how much facilitation results in successful outcomes, is yet to be thoroughly investigated [20–23]. Further, how to define or measure facilitation dose and content is unclear particularly because many studies use facilitation as part of a multifaceted implementation strategy. Recent findings from a case study of the Coordination Toolkit and Coaching Project which conceptualized facilitation intensity showed that intensity could be assessed quantitatively by the frequency and duration of facilitation encounters (dose) and qualitatively by the review of written facilitator reflections [22].

A multicomponent implementation strategy that comprises facilitation was particularly successful for evidence implementation in stroke care in the landmark Quality in Acute Stroke Care (QASC) Trial and translation studies [24–26]. Building on our previous research, the ongoing QASC Australasia Trial [27] is testing two different facilitation intensities or doses to support delivery of the Fever Sugar Swallow Protocols for stroke patients. As part of designing this trial, we undertook a scoping review to examine the evidence regarding how facilitation dose and content are described and reported in studies of evidence implementation in stroke intervention studies. Scoping reviews are well suited to clarifying concepts and definitions in a specific field as well as identifying key characteristics related to a concept [28, 29]. Guided by the Coordination Toolkit and Coaching Project’s quantitative measurement of facilitation dose, the specific research questions examined were:

1. Was facilitation dose (measured as the frequency and duration of facilitation encounters) reported in the included studies?
2. In what other ways, if any, was facilitation dose measured or reported in the included studies besides the frequency and duration of facilitation encounters?
3. What were the types of facilitation strategies (content) used to implement interventions in the included studies?
4. Did the included studies have reporting checklists or guidelines, and if so, which ones?

The findings from this scoping review will contribute to the body of knowledge on facilitation as an implementation strategy for evidence translation in healthcare settings.

## Methods

### Study design

This scoping review was conducted following the methodological framework described by Arksey and O’Malley which consists of five steps: (1) formulating the research question, (2) identifying relevant studies, (3) selecting eligible studies, (4) charting the data and (5) collating, summarising and reporting the results [30]. Reporting of the review complied with the Preferred Reporting Items for Systematic Reviews and Meta-Analyses – Scoping Reviews (Additional file 1) [31].

### Protocol and registration

The scoping review was registered with Open Science Framework (https://doi.org/10.17605/OSF.IO/WD5BJ).

### Eligibility criteria

Randomised controlled trials (RCTs) and quasi-experimental studies (non-randomised trial, pre-test and post-test [before-after], interrupted time series) [32] that evaluated facilitation (as defined by authors of included studies) as an implementation strategy to improve the uptake of stroke and/or transient ischemic attack (TIA) interventions were included in the review. Accompanying publications reporting on secondary outcomes (quantitative, qualitative, mixed methods or process evaluation papers) from eligible RCTs and quasi-experimental studies were also included if either the main results paper was unpublished, or the secondary outcomes papers provided relevant information not published in the main results paper. Studies were included if they were undertaken in acute and/or subacute care settings and evaluated interventions targeted at improving stroke and/or TIA management. Only peer-reviewed journal articles written in English language were included. Studies conducted in non-acute care settings only (e.g., primary health care, community clinics, nursing homes, pharmacies) were excluded. Grey literature such as theses/dissertations, conference abstracts, letters to editors, reports and guidelines were also excluded.

### Information sources

Electronic bibliographic databases Cochrane, CINAHL and MEDLINE were searched from January 2017 to March 2022 (with an updated search in July 2023), to identify eligible studies. The last two decades has seen considerable advancement in the field of implementation science, with a better understanding of implementation strategies [19]. The five-year search period was considered appropriate as research prior to this time was likely to demonstrate a lack of conceptual clarity on discrete implementation strategies, specifically inconsistency in the use of terminology and insufficient description of strategies [7].

### Search

The search strategy used different combinations of keywords and medical subject headings (MeSH) search terms with Boolean operators: facilitat* OR knowledge broker OR coach OR consultant OR mentor OR trainer OR implementation practitioner; intensity OR dose OR level OR amount OR type; implementation* OR dissemination; and intervention, quality improvement, and knowledge translation. The search terms were adapted for use with each electronic bibliographic database (Additional file 2). The reference lists of included papers were hand-searched for additional papers. The final search results were exported into EndNote X9.2 (Clarivate Analytics, Philadelphia).

### Selection of sources of evidence

The titles and abstracts of all papers retrieved from electronic databases and the additional papers identified from manual hand-searching were independently screened by one team member (HC) against relevance to the review questions and inclusion and exclusion criteria. After first-stage screening, the full texts of papers meeting the inclusion criteria were assessed by two team members (HC and OF) to determine the final studies included for analysis. The reference lists of the final included studies were also checked for relevant studies that could be included in the review. Disagreements regarding the study selection were resolved by a third member (SD).

### Data extraction

Data extraction was undertaken using Cochrane’s data collection form for RCTs and non-RCTs [33], which was adapted for the purpose of this review. Data extracted included: first author; year of publication; country of study; study participants; study setting; number of participants; study design; facilitation intensity or dose; mode of facilitation (internal, external, remote, or in-person); description of intervention and facilitation strategy; and study findings. Data extraction was performed independently by three research assistants with consensus on discrepancies undertaken by one team member (HC). Study authors were not contacted to identify additional information. Where available, additional information for the included studies were retrieved from their respective study protocols, clinical trial registrations, supplementary files and/or process evaluation papers to obtain detailed descriptions of the intervention and facilitation strategy. Study authors were not contacted for additional information.

### Critical appraisal of individual sources of evidence

The methodological quality or risk of bias of the included papers was determined. Critical appraisal of the papers was done by HC and a research assistant using the Mixed Methods Appraisal Tool [34], with consensus on discrepancies resolved by OF. Papers were assessed against five domains depending on the study design and the individual domains were rated as having either high, low, or unclear risk of bias.

### Synthesis of results

Narrative synthesis of the data was undertaken according to the Economic and Social Research Council’s guideline on the conduct of narrative synthesis [35]. To guide the analysis, the synthesis was structured around the four research questions. Preliminary synthesis involved the use of tabulation to enable data comparison across the different studies. In addition, textual descriptions of the studies were undertaken to summarise individual study findings and extract information relevant to the research questions. The characteristics of the included studies were explored to identify any similarity and/or differences in the studies in relation to the research questions.

## Results

**Fig. 1:**
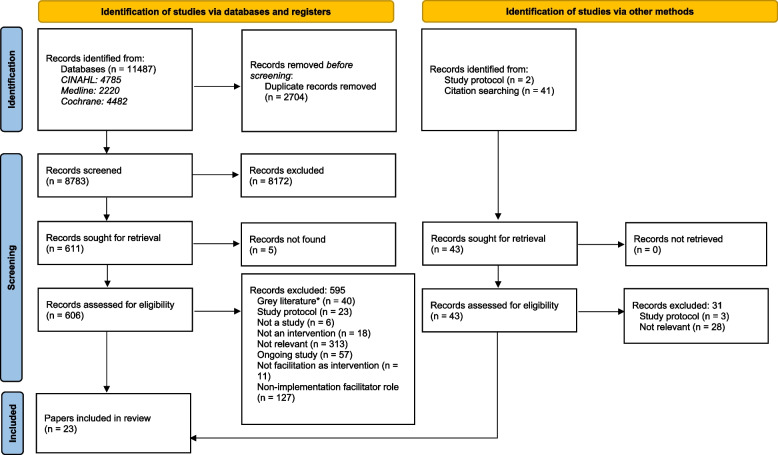
PRISMA diagram

A total of 8783 and 43 papers were identified in database and citation searches, respectively. After screening the titles and abstracts of papers against the inclusion and exclusion criteria, 649 full-text papers were assessed for inclusion. Of these, 23 papers from 10 studies were included in the analysis (Fig. 1).

### Characteristics of sources of evidence

Descriptive characteristics of the 10 studies and their accompanying papers are reported in Table 1. The study designs used in the included studies varied: three cluster RCTs [36–38] (the main study results of the Stroke Canada Optimization of Rehabilitation by Evidence Implementation Trial are yet to be published); two non-randomised controlled trials [39, 40]; and five pre-test and post-test (before-after) studies [41–45]. The accompanying papers from the ten studies also used a range of study designs, commonly mixed methods [46–48] and qualitative designs [49–51]. The main secondary outcomes evaluated in the accompanying papers were process evaluations [23, 38, 52, 53] and evaluations of stakeholder perspectives [47, 48, 50, 51, 54]. The 10 studies were conducted in the United States [39], Australia [36, 37, 40, 41, 45], Canada [38, 42, 43], and the Netherlands [44].

**Table 1:** Study characteristics

**Study name, publication(s), year, study design, outcome, country**	**Participants, setting, N**	**Mode of Facilitation (external, internal, remote, in-person) and dose (frequency, duration)**	**Description of intervention and facilitation strategy** **Comparator description**	**Original Study Findings**
Protocol-Guided Rapid Evaluation of Veterans Experiencing New Transient Neurological Symptoms (PREVENT) Bravata 2020; Non-randomised cluster trial, Intervention performance Bravata 2022, Mixed methods, Evaluation of QI sustainment Damush 2021a, Mixed methods, Evaluation of implementation bundle Damush 2021b, Mixed methods, Evaluation of intervention acceptability Penney 2021, Mixed methods, Stakeholder perception of external facilitation Rattray 2020, Observational qualitative, Evaluation of virtual “Hub” support of QI USA	Patients with inpatient treatment of TIA. 42 hospitals Intervention: N = 162 patients from 6 hospitals Control: N = 973 patients from 36 hospitals	External: research team, including QI nurse and QI physician Remote: facilitator support via phone, email or Skype In-person: in-person one-day kick-off meeting Facilitation dose: any interaction with external facilitator by phone, email, Skype, or in-person during one-year active facilitation period. Median of 24 facilitation episodes (mean of 8 education sessions, 10 quality process monitoring, 12 planning, 11 networking and 23 nurse external facilitator interactions) across 6 hospitals	Intervention: QI to improve TIA quality of care: quality of care reporting system, performance feedback, clinical progression, professional education, electronic health record tools, virtual collaborative. Online QI plans monitored in web-based audit and feedback system, with goal setting and monthly collaborative conferences for one year Facilitation strategy: external facilitation study team (primarily QI nurse and QI physician) supported education, team planning, goal setting; provided feedback; provided strategies for reflection and evaluation on practice One-day kick-off meeting, attended in-person or via teleconference, to develop targeted site-specific action plan with short- and long-term activities and plans Post-active implementation for one year, external facilitation no longer initiated by study team (sustainment) Comparator: matched CG site based on TIA patient volume, facility, and TIA quality of care^1^ For 6 IG sites: pre-stepped wedge allocation usual care	(1) At 1 year: increase in without-fail rate (patients received all 7 guideline-recommended TIA processes of care), p = 0.01 During sustainment: NS difference between groups (2) Increased measure of TIA quality (TIA processes of care received, as proportion of processes eligible), p = 0.01 During sustainment: NS difference between groups (3) NS change in anti-coagulation for atrial fibrillation (4) NS change in anti-thrombotic medication administered (5) NS change in brain imaging (6) NS change in carotid artery imaging (7) NS change in moderate/high potency statin therapy (8) NS change in HT control (9) NS change in neurological consultations (10) NS change in 90 d recurrent stroke rate (11) NS change in 90 d recurrent vascular event rate (12) NS change in 90 d mortality rate^1^ (13) In 6 IG hospitals: hospitals implemented 15–39 activities; mean 26.5 activities across sites. Pre-post difference NR (14) In 6 IG hospitals: increased team organisation score for improving TIA care, from 1 to 4 to 8, out of 10; mean 6.67 across sites. Between group differences NR (15) In 6 IG hospitals: 4 of 6 sites with ≥ 15 point improvement in without-fail rate (16) Variability in external facilitation activity: increased facilitation activity related to increased site implementation activity, or to prompt action during site inactivity^5^. Facilitators supported critical junctures of success/failure at two sites. Loss of external facilitation support at sustainment led to decreased motivation, team activity and coordination, if PREVENT not implemented into routine practice
Stroke123 Cadilhac 2019, Prospective before-and-after study, Intervention effectiveness Thayabaranathan 2021, Process evaluation of external facilitator role Australia	Adult patients admitted for stroke or TIA. 19 hospitals Intervention: N = 2682 Control: N = 5596	External: external staff with nursing or allied health QI officer Remote: Telephone or email support In-person: in-person 1 × 3 h workshop. Face-to-face peer support after workshop as needed Facilitation dose: 1 × 3 h on-site workshop; ongoing phone, email or face-to-face support. Median 22/mean 30 h of external facilitator support across all hospitals	Intervention: organisational INT to improve quality of stroke care, with 2 stages: financial incentives to establish or enhance stroke units (time 1), financial incentives with QI external facilitator (time 2) Facilitation strategy: external facilitator delivered workshop to review hospital performance, provided site-specific strategies, developed action plan using PDSA to improve clinical guideline adherence. Ongoing peer support, via phone, email or face-to-face. Additional support for remote education, networking and shared learning Comparator: Pre-INT usual care	(1) Increased composite score (received all 8 guideline-recommended stroke processes of care) post-QI facilitation stage (post-time 2), p < 0.001. After adjustment of patient confounders, p = 0.007 Increased composite score post-INT (baseline to 13 mo post-INT), p < 0.001. After adjustment of patient confounders, p = 0.005 (2) Increased patients treated in stroke unit over time (baseline to 13 mo post-INT), p < 0.001 (3) NS difference in receiving thrombolysis for ischemic stroke (4) Increased patients with early mobilisation over time (baseline to 13 mo post-INT), p < 0.001 (5) Increased swallow screening or assessment over time (baseline to 13 mo post-INT), p < 0.001 (6) Increased early aspirin delivery over time (baseline to 13 mo post-INT), p = 0.047 (7) Increased discharge with anti-HT medication over time (baseline to 13 mo post-INT), p < 0.001 (8) Increased discharge with anti-thrombotic medication over time (baseline to 13 mo post-INT), p < 0.001 (9) Increased discharge to community with care plan over time (baseline to 13 mo post-INT), p < 0.001 (10) No association between amount of external facilitation and absolute change in composite score. 13 of 19 hospitals with increased mean composite score^8^. 9 of 18 hospitals with increased absolute change in composite score
Shared Team Efforts Leading to Adherence Results (STELAR) Cadilhac 2022, Stepped-wedge cluster RCT, Intervention effectiveness Cadilhac 2017, Mixed-methods controlled before–after observational study, Pilot test of intervention Australia	Adult patients admitted for stroke or TIA Intervention RCT: N = 9 hospitals, 2146 patients Control RCT: N = 9 hospitals, 1001 patients Intervention pilot: N = 2 hospitals, 438 episodes of care Control pilot: N = 2 hospitals, 419 episodes of care	External: research team, including implementation researcher and nurse researcher Remote: Telephone or email support In-person: 1 × 2 h workshop Facilitation dose: 1 × 1 h videoconference, 1 × 2 h in-person workshop, ongoing phone or email support for 2 mo	Intervention: two-stage INT to improve adherence to acute stroke care clinical indicators. Audit with feedback; tailored site-specific action plan; nomination of clinical champion Facilitation strategy: external facilitators delivered two workshops for audit feedback, education on clinical guideline adherence, design of site-specific action plan. Ongoing remote support via telephone or email Comparator: Pre-INT usual care	(1) RCT: increased composite score for all site-specific plan on guideline-recommended stroke processes of care [below] post-INT, p = 0.016 Pilot: increased composite score post-INT, p < 0.001^10^. Sustained improvement at one year post-INT sustainment, p = 0.08 (2) RCT: increased odds of treatment in stroke unit, p < 0.05 (3) RCT: NS difference in swallow screening or assessment before oral intake (4) RCT: NS difference in early mobilisation (5) RCT: NS difference in discharge with anti-HT medication (6) RCT: increased odds of discharge with anti-HT medication, p < 0.05 Pilot: increased odds of discharge with anti-HT medication, p = 0.001. NS difference at one year post-INT sustainment (7) RCT: increased odds of discharge with care plan, p < 0.05 Pilot: increased odds of discharge with care plan, p < 0.001^10^. NS difference at one year post-INT sustainment (8) RCT: NS difference in receiving thrombolysis, for ischemic stroke (9) RCT: NS difference in receiving thrombolysis at ≤ 60 min of admission, for ischemic stroke (10) RCT: NS difference in receiving anti-platelet medication at ≤ 48 h of admission, for ischemic stroke (11) RCT: NS difference in discharge with lipid-lowering medication, for ischemic stroke (12) RCT: NS difference in 30 d mortality rate (13) RCT: NS difference in 90 d mortality rate (14) RCT: NS difference in disability status at 90–180 d follow up
Jolliffe 2020, Non-randomised three-arm cluster-controlled feasibility study, Feasibility, efficacy and acceptability of two implementation packages Australia	Stroke patients with upper limb stroke rehabilitation with OT or PT. 6 hospital ward or community neurological rehabilitation sites Intervention A: N = 20 Intervention B: N = 17 Control: N = 18	External: research team Remote: Intervention delivered via Trello for group B and participants were encouraged to post any comments/questions to the research team In-person: 6 × 45 min face-to-face education sessions and fortnightly/monthly coaching/mentoring for Intervention group A Facilitation dose: fortnightly/monthly coaching with facilitator; and 6 × 45 min face to face education, for 3 mo	Intervention group A: Facilitator-mediated implementation of rehabilitation guidelines. Includes point of care videos, face to face education, online education modules, written manuals, fortnightly/monthly coaching and mentoring, auditing and feedback, access to physical resources, environmental alterations for encourage patient practice Intervention group B: self-directed implementation of rehabilitation guidelines. Includes point of care videos, online education modules, posters with guideline recommendations, written manuals, physical resources, patient handouts. INT content delivered online Comparator: usual care	(1) Increased adherence to rehabilitation OT/PT guideline: IG 1 vs IG 2, p < 0.0001; IG 1 vs CG, p < 0.0001; NS IG 2 vs CG (2) NS change in Box and Block Test, between IG 1 vs IG 2, IG 1 vs CG, IG 2 vs CG (3) Improved Fugl-Meyer Upper Extremity Assessment upper limb outcomes in IG 2 vs. CG, p = 0.027. NS between IG 1 vs IG 2, and IG 1 vs CG (4) NS change in self-reported minutes of weekly therapy, between IG 1 vs IG 2, IG 2 vs CG, IG2 vs CG
Carers Count Levy 2022, Interventional study and mixed methods evaluation, Intervention development and implementation Australia	Stroke survivors and carers. 1 stroke rehabilitation ward N = 30 stroke survivors, 30 carers	External: research team In-person: fortnightly meetings and ongoing staff training Facilitation dose: fortnightly meetings with team members and management; and ongoing staff training, for 5 mo	Intervention: Facilitation of new exercise-based group of stroke survivors and carers External facilitator: fortnightly meetings with allied health team. Conducted on-going allied health staff training. Tailored INT to address barriers. Developed implementation scale-up, education resources, manuals. Involved consumer representatives in planning and implementation. Supported electronic medical record data collection Comparator: pre-trial usual care	Post-trial only, focused on INT instead of facilitator involvement (1) ≥ 80% agreement on group attendance benefits, satisfaction with staff support in group, understanding of post-stroke physical ability, confidence in post-discharge stroke management, socialisation with group peers (2) 100% agreement on exercise-based group not increasing post-stroke stressors (3) Qualitative outcomes: exercise-based group as ‘something to look forward to’, positive shared experience by stroke survivor and carer, decreased isolation, perceived benefits
Moore 2020, Pre-post study, Evaluation of intervention implementation, sustainability and clinician adherence Canada	Subacute stroke patients with goal to improve walking. 1 subacute inpatient stroke rehabilitation facility N = 157 patients	External: research team – university and translational researchers Remote: 5–60 min monthly phone conversations for 15 mo In-person: 1 × 3 h + 1 × 1 h education sessions for stroke staff; 5 × 1.5 h non-core stroke staff training education sessions Facilitation dose: various knowledge translation interventions, including weekly team meetings, for 12 mo; education to stroke team and non-core staff; phone call/s	Intervention: multicomponent, iterative implementation plan with facilitation, leadership and knowledge translation interventions, to implement a gait assessment battery into routine clinical practice Facilitators: researchers, with clinical team, identified site-specific barriers and enablers, then co-designed tailored knowledge translation INT, with education, leadership support, process changes, audit and feedback, equipment purchase, environment modification. Weekly feedback in team meetings. Phone calls to problem solve, discuss adherence, barriers and knowledge translation INTs Comparator: pre-trial usual care	(1) Increased adherence to Gait and Balance assessment, from 46% at baseline to 85% at 6 mo and 95.2% at 48 mo. Significance NR (2) Increased use of 10 min walk test, p = 0.03
Stroke Canada Optimization of Rehabilitation by Evidence-Implementation Trial (SCORE-IT) Munce 2017, Qualitative descriptive, Stakeholder evaluation of barriers and facilitators Salbach 2017, Quantitative process evaluation of SCORE-IT Canada NB: main trial results of SCORE-IT have not been published	Stroke rehabilitation patients. 20 sites Intervention: N = 9 sites, 169 patients Control: N = 8 sites, 143 patients	Internal: nurse and physical therapist External: research team Remote: teleconference, by external facilitators for internal facilitators In-person: internal facilitators on-site, 4 h/wk for 16 mo Facilitation dose: 1 × 2d training workshop for internal facilitators; teleconference support for internal facilitators; internal facilitator on-site for 4 h/wk for 16 mo	Intervention: facilitated knowledge translation INT, with internal facilitators at each INT site for 4 h/wk Internal facilitator: promoted guideline implementation at INT site. Received two-day workshop, including change management, implementation strategies, developing implementation plan to address barriers, and incorporating behaviour change strategies. Contacted other facilitators via teleconference and web-based platform to share successful implementation strategies Stroke teams provided with stroke rehabilitation guideline, evidence-based treatment protocols, posters, reminder cards External facilitator: research team provided advice and support to internal facilitators via teleconference Comparator: passive knowledge translation INT Received stroke rehabilitation guideline without treatment protocols, handbook, and educational DVD on post-stroke standardised assessment tools. Opt-in facilitator role by motivated staff members	(1) Facilitator staff as leaders/champions supported implementation and provided continuity for trial procedures/tasks in face of high staff turnover. Lack of access to facilitator staff was a barrier: lack of champion, impeded continuity in face of staff turnover, no sustainability for knowledge translation interventions (2) Increased implementation of sit-to-stand training, p = 0.028, and walking practice, p = 0.043, in IG. Increased implementation of standing balance training, p = 0.037, in CG. Decreased implementation of stretching training and sitting balance training in IG, p < 0.05
Implementation of Partners of Aphasic clients Conversation Training (ImPACT) Wielaert 2018, Interventional study with mixed methods evaluation, Evaluation of intervention uptake, barriers/facilitators and implementation methods Wielaert 2017, Qualitative, Exploration of client and stakeholder experiences Wielaert 2016, Pre-post study, Assessment of stakeholder eligibility The Netherlands	Partners of patients with aphasia. 7 rehabilitation sites and 3 nursing homes N = 10 sites	Internal: speech language therapists, 2 per site External: research team Remote: telephone consultations, phone contact, email contact, quarterly newsletters In-person: 4 × 1d meetings, 2 × 2 h outreach visits, 1 × 1d outreach meeting to site Facilitation dose: 4 × 1d meetings and 2 × 2 h outreach visits for internal facilitators, by external facilitators. 1 × outreach meeting to site by external facilitator. Telephone consultations, phone and email contact, quarterly newsletters, for internal facilitators by external facilitators, across 13 mo	Intervention: multicomponent implementation INT of conversation partner training in aphasia (PACT). Financial support for local coordinators; internal facilitator training; aphasia-friendly education materials; feedback on recruitment, PACT training, implementation issues; phone and email reminders with newsletters Internal facilitators: implemented PACT on-site – advocated for PACT with multidisciplinary team, provided and engaged clients in PACT. Two meetings for skill training in PACT. Two meetings on developing implementation plans, including goal setting and PACT analysis External facilitators: trained internal facilitators in 4 meetings. Delivered three outreach visits – 2 for internal facilitators, 1 for multidisciplinary team and manager. Phone and email PACT supervision for internal facilitators after 4th meeting Comparator: pre-trial usual care	(1) 7 centres with uptake of PACT in care pathway at 8mo post-INT (2) Components necessarily for INT implementation: financial support; education to deliver PACT for internal facilitators; outreach visit and PACT presentation by external facilitator for multidisciplinary team and manager
iWalk study Salbach 2022a, Before-and-after study, iWalk toolkit evaluation Salbach 2022b, Mixed methods process evaluation of intervention implementation Salbach 2021, Realist evaluation, Site-specific context-mechanism-outcome synthesis Canada	Physical therapists. 9 hospitals Intervention: N = 375 hospital visits, 33 physical therapists, 7 practice leaders Control: N = 347 hospital visits	Internal: physical therapist, manager or practice leader providing post-stroke acute or rehabilitation care External: physical therapist expert on the research team Remote: email or phone contact In-person: 3 × 1 h learning sessions by internal facilitator Facilitation dose: 3 × 1 h learning sessions by internal facilitator; and email or phone support from external facilitator, over 21 mo	Intervention: implementation of iWalk toolkit with 10 m walk test and 6 min walk test in stroke rehabilitation. Toolkit: educational guide, educational video, mobile app Internal facilitator: set up walkways for walk tests, organised and facilitated 3 learning sessions within a 5-month period External facilitator: email or phone support Comparator: pre-trial usual care	(1) Lack of internal facilitator in non-neurology services (e.g. palliative care, emergency care) did not have authority to implement iWalk toolkit: no walk test walkways, no motivation to attend learning sessions (2) In acute care: internal facilitators reminded physical therapists to use iWalk toolkit and coordinated learning sessions. Internal facilitators being involved in clinical practice gave them more influence over physical therapists (3) In rehabilitation care: internal facilitators adapted learning session materials, offered off-site attendance by teleconference, or organised on-site training for off-site therapists (4) Internal facilitators supported daily interaction with physical therapists as reminders of iWalk toolkit use; addressed local policy that limited walkway set up; adapted recommended practice to local context, patient population and negative outcome expectations (5) Increased administration of 10 m walk test, p < 0.05 (6) Increased administration of 6 min walk test, p < 0.05 (7) Increased odds of administering 10 m walk test once during hospital stay, p < 0.05 (8) Increased odds of administering of 6 min walk test once during hospital stay, p < 0.05
Triage, treatment and transfer of patients with stroke in emergency department trial (the T^3^ Trial) Middleton 2019, Cluster RCT, Intervention effectiveness McInnes 2020, Qualitative process evaluation of factors influencing protocol uptake Australia	Stroke patients admitted to ED. 26 EDs with stroke units Intervention: N = 13 EDs, 677 patients Control: N = 13 EDs, 920 patients	Internal: site clinical champion, as local opinion leader External: nurse researcher Remote: teleconference, phone and email follow up In-person: 2 × 1 h workshops; 1 × 30 min education session; engagement every 6 wks, alternating between site visit and remote teleconference call Facilitation dose: 2 × 1 h face-to-face workshops to identify barriers to implementation and develop action plans, 1 × 30 min education session. Engagement every 6 wks, alternating between site visit and teleconference call every 3 mo. Follow up of internal facilitator-initiated emails and phone calls	Intervention: assess stroke patients for thrombolysis eligibility; treat with thrombolysis; monitor fever, BGL and swallow function; transfer to stroke unit. Delivered via workshops, clinician education, reminders (lanyards, ED posters) and site engagement with visits, telephone and email External facilitator: led workshops with clinicians; supported development of site-specific action plan; delivered education to clinicians and clinical champions; maintained site engagement Internal facilitator: led local clinical change; delivered education for clinicians and new staff Comparator: usual care	(1) NS difference in death or functional dependency status at 90 d. After adjustment for stroke onset to ED admission time, NS (2) NS difference in functional dependency status at 90 d (3) NS difference in health status at 90 d (4) NS difference in patients screened for thrombolysis eligibility (5) NS difference in receiving thrombolysis for ischemic stroke (6) NS difference in 4-hrly temperature monitoring in ED (7) NS difference in 6-hrly BGL monitoring in ED (8) NS difference in swallow screening or assessment in 24 h of ED admission (9) NS difference in transfer to stroke unit within 4 h of admission (10) Internal facilitator needed to unite ED and stroke clinicians and lead implementation. Lack of authority of, or respect towards, internal facilitator as barrier to medical staff change (11) Internal facilitator, as nurses, reported lack of medical staff in internal facilitator roles as barrier to engaging medical staff in implementation and practice change

*BGL* blood glucose level, *d* day/s, *hr* hour/s, *ED* emergency department, *HT* hypertension, *INT* intervention, *min* minute/s, *mo* month/s, *NR* not reported, *NS* non-significant, *OT* occupational therapist, *PT* physical therapist/physiotherapist, *QI* quality improvement, *RCT* randomised controlled trial, *TIA* transient ischemic attack, *wk* week/s

Different stroke and/or TIA interventions were evaluated. Six studies involved stroke rehabilitation interventions [38, 40, 42–45]. One study [37] focused on stroke intervention implementation in the emergency department while the remaining three studies involved interventions aimed at improving the quality of stroke or TIA inpatient care [36, 39, 41]. Of the 10 studies, seven evaluated interventions in patients with TIA [39], stroke [37, 38, 40, 42], and either stroke or TIA [36, 41]. The remaining three studies involved stroke survivors and carers [44, 45] and physical therapists [43].

The patient and clinical process of care outcomes reported in the studies varied. However, there were a few studies that reported similar outcomes, such as guideline-based stroke or TIA processes of care [23, 36, 47, 53]; administration of rehabilitation walk tests [42, 52]; and mortality at 90 days post-discharge [36, 37, 39].

### Facilitation dose reporting and measurement

Table 1 provides information on the facilitation dose reported in the included studies. Of the 10 studies, only two reported on the total facilitation dose, quantitatively measured as the frequency and duration of facilitation encounters or activities such as education delivery, coaching, training, barrier and enabler identification, action plan development and data collection,

Thayabaranathan et al. [23] reported the amount of external facilitation as ‘the frequency and duration of professional behaviour change support provided to clinicians, mode of support delivery, and time spent delivering support’ [23]. This was measured for two implementation strategies: one 3-h face-to-face workshop to develop an implementation strategy action plan to improve stroke care, and ongoing phone, email, or face-to-face support. There was a mean of 30 h (standard deviation [SD]: 14) of total facilitation time for the 19 participating hospitals, constituting a mean of 19 h (SD: 11) of face-to-face contact, 5 h (SD: 2) of phone contact, and 7 h (SD: 4) of email contact. There was a clinically significant, but not statistically significant, difference in hours of facilitation time between the 14 hospitals with an implementation strategy action plan (mean: 32, SD: 15) and the 5 hospitals which did not develop an implementation strategy action plan (mean: 25, SD: 10).

Damush et al. [47] defined facilitation dose as interactions between the site team members and external facilitator (a quality improvement nurse or physician) by phone, email, Skype teleconference, or in-person. These interactions (referred to as episodes) were frequently contained in a single email chain but could extend over several weeks and were regarding a specific request, problem, or question. The facilitation dose was measured for the six participating sites with each site receiving a mean of 24 episodes of external facilitation before and during the one-year active implementation period [48]. The facilitation dose (episodes) was also measured for specific activities performed by the external facilitator namely education (mean: 8), quality process monitoring (mean: 10), planning (mean: 12) and networking (mean: 11) [47].

Authors of the remaining eight studies reported on only the frequency and/or duration of individual facilitation encounters with no combined measurement of facilitation dose. For example, the frequency and duration of education sessions or workshops conducted by the facilitator were reported in some of the included studies: one one-hour videoconference and one two-hour in-person workshop [36]; one two-day workshop [38]; and three learning sessions [55].

Authors of several studies also reported on staff support time. This was sometimes measured by frequency and duration, for example four hours of internal facilitator support per week for 16 months [38]; or measured only by duration, for example email or phone support by external facilitator for 21 months [43, 55]; or phone and email contact, newsletters and phone consultations by external facilitators for 13 months [44].

Mentoring, coaching sessions and team meetings by the facilitator were either reported by frequency and/or duration. For example, fortnightly or monthly coaching with external facilitator across three months [40], weekly team meetings for 12 months [42], and fortnightly team meetings with ongoing staff training for five months [45].

While Middleton et al. [37] inferred that the regular provision of ongoing intensive structured support to clinical champions by an external facilitator represented facilitation dose, there was no overall quantitative measure of facilitation dose reported in the study. The intensive structured support comprised sustained engagement with direct contact every six weeks (alternating between site visits and teleconferences every three months), facilitation of two one-hour face-to-face multidisciplinary team workshops, and a 30-min education session at each site.

### Facilitation activities and facilitator roles

Although the type of facilitation strategies used to implement stroke and/or TIA interventions were broad, there were similarities between the studies. All studies used external facilitators who either supported implementation of the intervention [36, 40, 41, 45, 47] or supported internal facilitators [44, 50, 55] to implement some or all aspects of the intervention. In the study by Middleton et al. [37], external facilitators performed both roles, while in the study by Moore et al. [42], external facilitators worked collaboratively with the clinical team and leadership to co-create and implement a knowledge translation intervention.

Four studies used internal facilitators, such as local clinicians [37, 43, 44, 50]. Nine studies used remote facilitation via telecommunication (phone calls, emails, teleconferences) [36–39, 41–44] or online tools (comments/questions to the research team via Trello) [40]. The studies also used in-person facilitation, such as in-person facilitation of kick-off or planning meetings [36, 37, 39, 41, 44], in-person facilitation of education sessions or workshops [37, 40, 42, 43, 45] or site/outreach visits [37, 44]. All studies combined different modes of facilitation: external and in-person [45]; external, remote and in-person [36, 39–41]; internal, external and in-person [42]; internal, external, remote and in-person [37, 38, 43, 44].

Facilitator roles varied and there was an overlap of roles between studies. Roles involved individualised and site-specific briefing, set up, action planning and/or goal setting; problem-solving; or supporting clinicians to identify enablers and barriers to change [36–42, 44, 45]. Facilitators delivered coaching, training, education, progress feedback, or ongoing support; or provided consultations [36–45]. Facilitators also undertook site visits as well as monitored and collected data for research or quality improvement processes [37, 44, 45]. Only two studies involved facilitators assisting with the development of implementation resources [40, 45].

### Reporting checklists/guidelines

Eight papers [36, 37, 39, 41, 46, 48, 55, 56] from five studies had guidelines or checklists, but only two papers [36, 48] provided completed checklists as supplementary material (Table 2). One paper [55] had the TIDier guideline and three [39, 46, 48] had the Standards for Reporting Implementation Studies (StaRI) statement, which are intended to support researchers to describe interventions and implementation studies in sufficient detail to enable replication. One paper [36] had the Consolidated Standards of Reporting Trials (CONSORT) statement which aims to improve reporting of RCTs. One paper [37] used the Standard Protocol Items: Recommendations for Interventional Trials (SPIRIT) statement in the trial protocol, which enhances clinical trial protocol reporting. One paper [56] had the Standards for QUality Improvement Reporting Excellence (SQUIRE) statement which guides the reporting of system-level initiatives to improve healthcare quality.

**Table 2:** Guidelines and checklists used in the included studies

Study name	Publication(s)	Guideline/ checklist used	Guideline/checklist included as an appendix in publication
Protocol-Guided Rapid Evaluation of Veterans Experiencing New Transient Neurological Symptoms (PREVENT)	Bravata 2020 Bravata 2022 Damush 2021a Damush 2021b Penney 2021 Rattray 2020	StaRI STROBE	Yes – StaRI No – STROBE
Stroke123	Cadilhac 2019 Thayabaranathan 2021	STROBE	No
Shared Team Efforts Leading to Adherence Results (STELAR)	Cadilhac 2022 Cadilhac 2017	CONSORT SQUIRE	Yes – CONSORT No – SQUIRE
-	Jolliffe 2020	-	-
Carers Count	Levy 2022	-	-
-	Moore 2020	-	-
Stroke Canada Optimization of Rehabilitation by Evidence-Implementation Trial (SCORE-IT)	Munce 2017 Salbach 2017	-	-
Partners of Aphasic clients Conversation Training (imPACT)	Wielaert 2018 Wielaert 2017 Wielaert 2016	-	-
iWalk	Salbach 2022a Salbach 2022b Salbach 2021	RAMSES SRQR TIDier	No
Triage, treatment and transfer of patients with stroke in emergency department trial (T^3^ Trial)	Middleton 2019 McInnes 2020	SPIRIT	Yes [protocol]

*CONSORT* Consolidated Standards of Reporting Trials, *RAMSES* Realist And Meta-narrative Evidence Syntheses: Evolving Standards, *RCT* Randomised Controlled Trial, *SPIRIT* Standard Protocol Items: Recommendations for Interventional Trials, *SQUIRE* Standards for QUality Improvement Reporting Excellence, *SRQR* Standards for Reporting Qualitative Research, *StaRI* Standards for Reporting Implementation Studies, *STROBE* Strengthening the Reporting of Observational Studies in Epidemiology, *TIDier* Template for Intervention Description and Replication

### Critical appraisal within sources of evidence

**Fig. 2:**
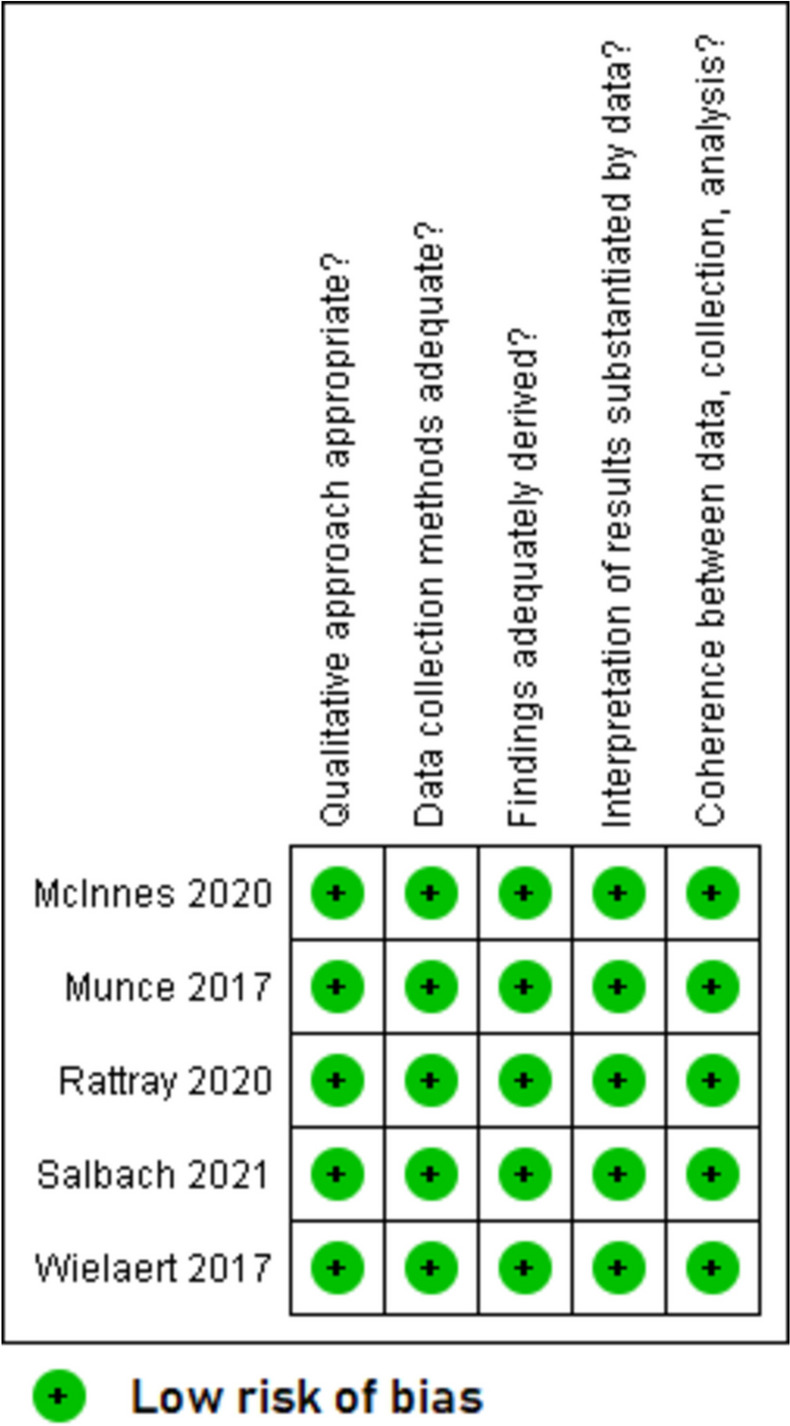
Critical appraisal of qualitative papers

**Fig. 3:**
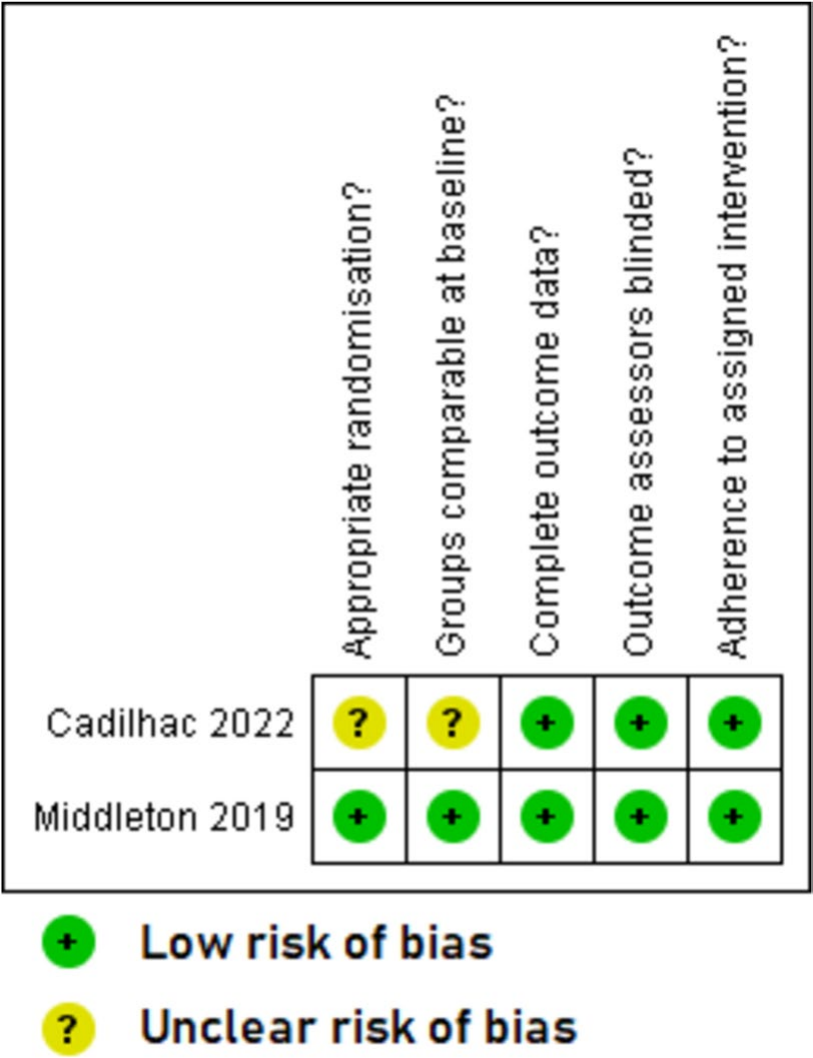
Critical appraisal of randomised controlled trials

**Fig. 4:**
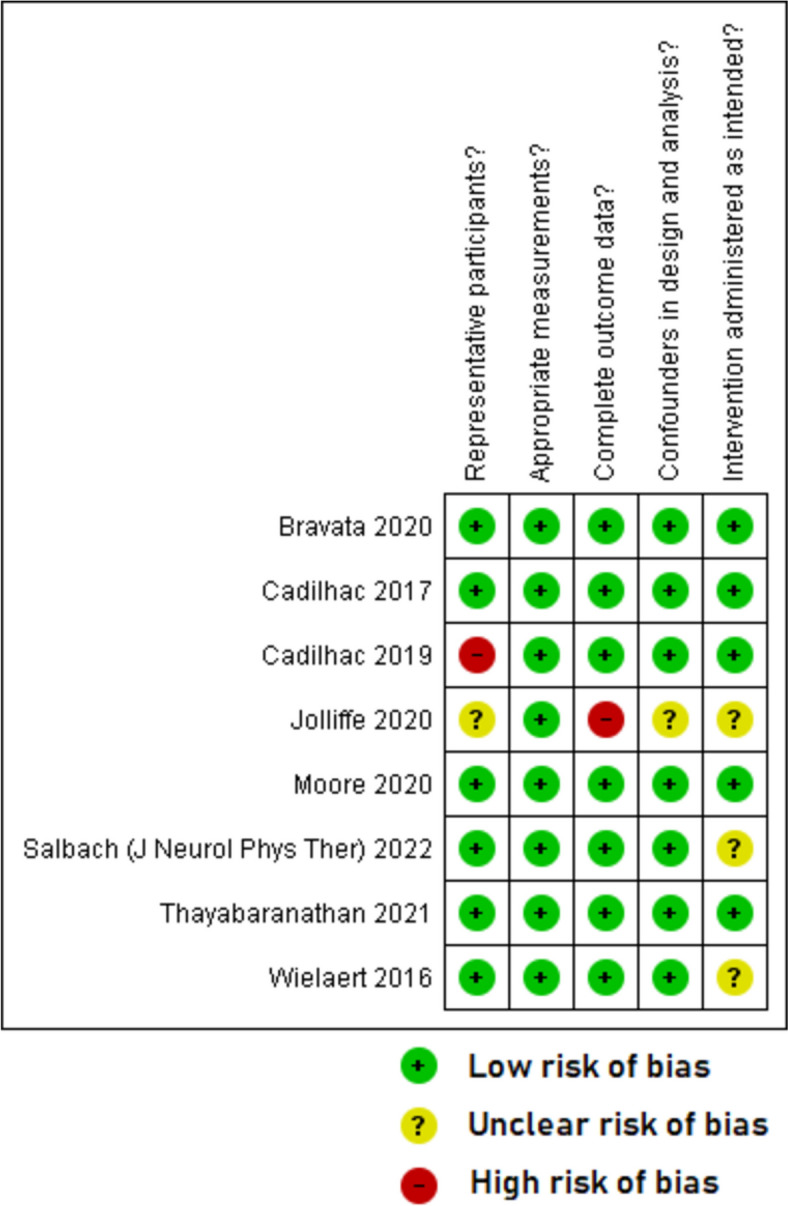
Critical appraisal of quantitative non-randomised papers

**Fig. 5:**
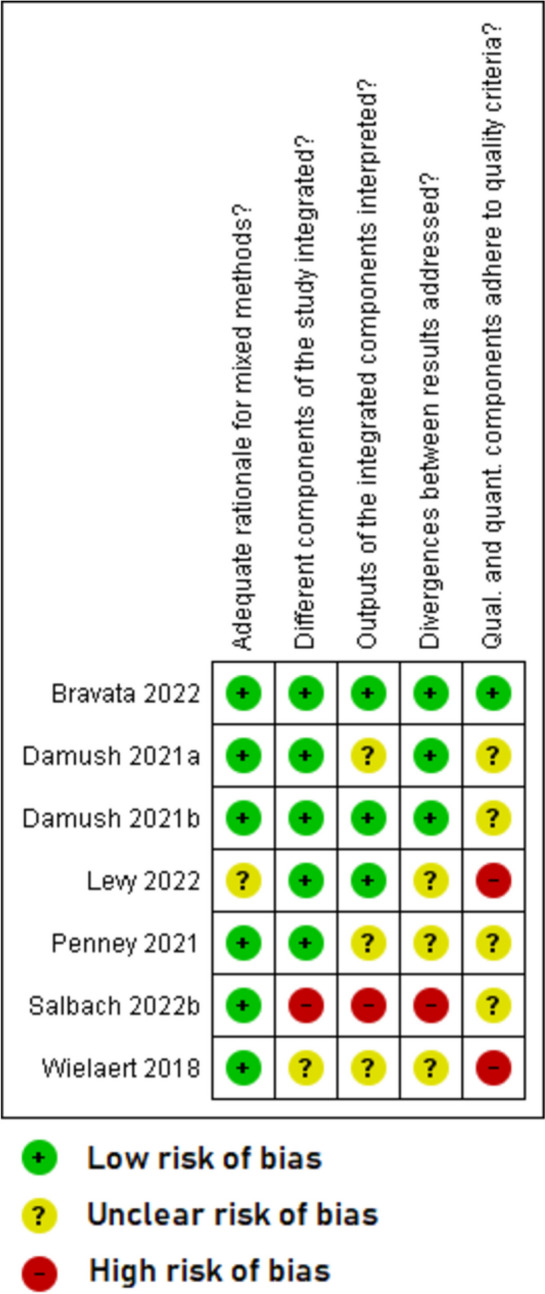
Critical appraisal of mixed methods papers

**Fig. 6:**
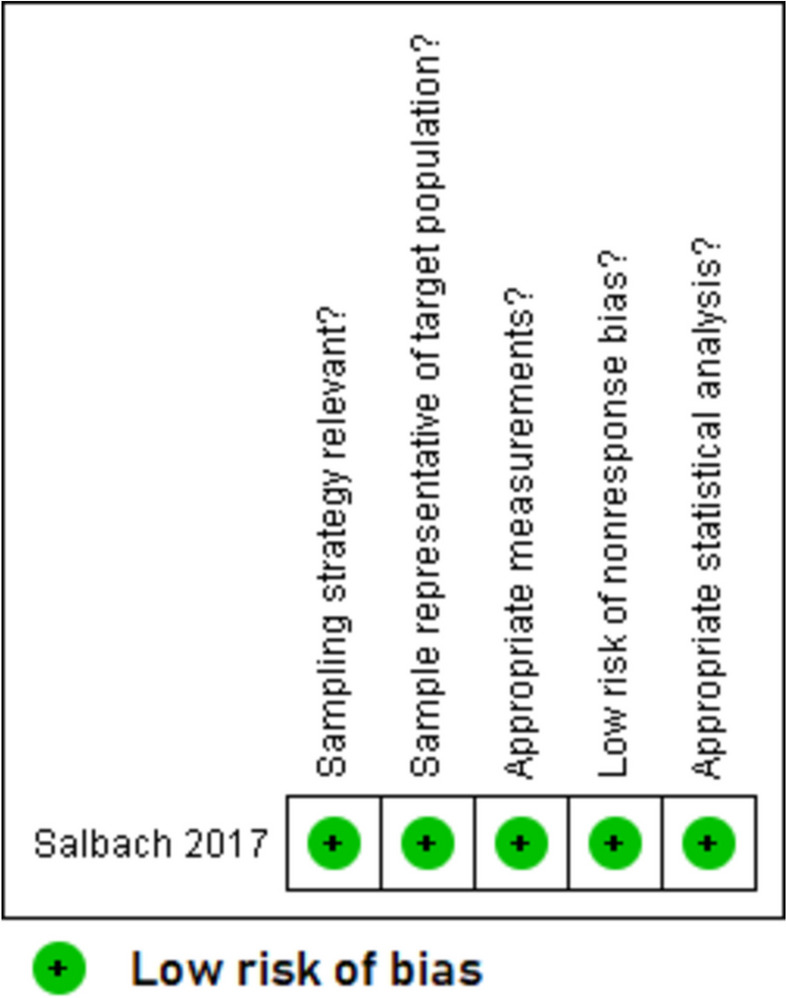
Critical appraisal of quantitative descriptive paper

Overall, the quality of included studies was mixed. The five qualitative papers [49–51, 53, 55] had a low risk of bias for all the five domains that were assessed (Fig. 2). The two RCTs with published trial results [36, 37] (Fig. 3) and the eight quantitative non-randomised papers [23, 39–43, 54, 56] (Fig. 4) generally had a low or unclear risk of bias for the domains assessed. Three of the seven mixed methods papers had a high risk of bias for the domains of adequately integrating quantitative and qualitative data, explaining where divergence in quantitative and qualitative data occurred, and overall quantitative and qualitative data quality [44, 45, 52]. The remaining four mixed methods papers [46–48] had a low or unclear risk of bias for the domains of rationalising the used of mixed methods, integrating quantitative and qualitative data, and overall quantitative and qualitative data quality (Fig. 5). The quantitative descriptive paper [38] had a low risk of bias for all the domains (Fig. 6).

## Discussion

We examined the concepts of facilitation dose and content within the context of implementation of stroke and/or TIA interventions. Our findings revealed a significant gap in the literature regarding both the measurement and reporting of facilitation dose and content. Only two of the 10 studies measured the total facilitation dose while the remaining eight studies reported only on different facilitation encounters/activities with no overall measurement of facilitation dose. We found that the content of the facilitation strategies was broad with an overlap between studies in the roles performed by facilitators. In addition, there was a minimal use of reporting checklists/guidelines, particularly those intended to support researchers to describe interventions and implementation studies in sufficient detail to enable replication.

A notable finding from this scoping review was the lack of a standardised method for measuring facilitation dose in the included stroke/TIA studies. While some studies measured an overall dose for all facilitation encounters or activities, others only self-reported individual facilitation encounters without any quantitative measures. Furthermore, some studies measured the facilitation duration without the frequency and vice versa. Our measurement of facilitation dose was guided by the Coordination Toolkit and Coaching Project which was based on both the frequency and duration of facilitation encounters [22]. However, this measurement approach is limited by using only time as a measure for dose. While time is clearly one dimension of dose, there are other dimensions which might modify the effectiveness of facilitation that are important to also measure. For example, 8 h of didactic sessions are not the same as 8 h of interactive sessions and measuring only the time spent may not account for variability in engagement and other context-dependent factors. There has been a recent call for research across healthcare services to develop measures that assess facilitation intensity beyond its frequency and duration, and take into account the energy (mental, emotional, physical) expended by implementation facilitators during each activity and cumulatively across activities [22].

It is important to note that measurement of the true dose or intensity of facilitation may be challenging as facilitation is a complex multifaced concept which encompasses a broad range of techniques and strategies needed to bring about intervention implementation success [6, 22, 57]. Further, facilitation may take different forms such as internal facilitation (facilitation by existing staff within the implementation site), external facilitation (facilitation by a person external to the implementation site), or a combination of both [6]. As a result, there is the likelihood that a considerable amount of facilitation occurs outside the defined prescribed role, particularly for internal facilitation. For example, when a staff member employed in a clinical leadership role that involves implementing practice change has to also perform internal facilitator duties for a quality improvement initiative. The external facilitator role is usually better delineated, as this is typically a dedicated role undertaken by an individual employed by an external organisation [6] and may therefore be easier to measure. In addition, factors such as the specific objectives of the intervention, the interpersonal and communication skills, and the familiarity with local processes and culture of the facilitator [58] may have a potential impact on the facilitation process. Therefore, the true facilitation dose may not always be easily and consistently measured or accurately estimated.

To better understand implementation strategies such as facilitation, it is also essential to delve deeper into the content or elements that make up the facilitation strategies. We found that the facilitation activities and roles of facilitators in the included studies were broad, which is consistent with the implementation science literature [6, 8, 9, 20, 59]. Further, facilitation was used in all the studies as part of a multicomponent implementation strategy. Therefore, the facilitator role often involved the incorporation or use of other implementation strategies to provide support for intervention delivery such as conducting educational meetings, outreach visits, training, providing reminders, or undertaking audit and feedback. This complex multifaceted nature of facilitation reflects the diversity of approaches in getting evidence into practice [20, 57]. This complexity also contributes to the challenges of clearly describing, operationalising and measuring facilitation [18].

Process evaluations aim to provide insight into the context in which implementation strategies such as facilitation are applied in real-world settings [60, 61]. They are able to describe in detail how the strategy was developed, delivered, participants exposure and their experience with the implementation activities that make up the strategy, as well as the contextual factors impacting on the strategy [61]. Evidence shows that facilitation activities tend to occur flexibly in response to local circumstances with the ever-evolving context dictating the intensity of most facilitation activities [62]. Furthermore, findings from the process evaluation of a trial which evaluated two facilitation doses with no significant difference between them [21] revealed that the facilitation types were unable to overcome the influence of contextual factors such as limited resources and lack of managerial and staff support [63]. This shows that there are factors which impact on facilitation that cannot necessarily be quantified and are better understood through concurrently undertaken process evaluations.

Our findings also illustrate the lack of reporting on specific details of intervention delivery thereby highlighting the importance of transparent reporting practices in research [64, 65]. Only eight papers [36, 37, 39, 41, 46, 48, 55, 56] from five studies reported using guidelines or checklists, of which four papers [39, 46, 48, 55] from two studies used either the TIDIer guideline or StaRI statement which aim to support researchers to describe interventions and implementation studies in sufficient detail to enable replication. Unsurprisingly, three of these four papers had a low risk of bias across the assessment domains, indicating that studies which adhere to reporting guidelines for implementation studies may be more likely to be reported precisely. Powell et al. [19] noted that implementation strategies are often poorly described in study protocols or empirical studies. This has the potential to limit the reproducibility of research as well as the interpretation of study findings [18, 66].

Another important implication of our findings is the potential impact of facilitation on intervention effectiveness and efficiency. Knowledge of the optimal facilitation dose and type of facilitation strategies required to achieve desired outcomes is important for designing effective interventions and allocating resources efficiently. If researchers are unable to draw meaningful conclusions regarding the impact of facilitation on study outcomes due to inadequate measurement and reporting, they may erroneously attribute differences in outcomes solely to the intervention. This could result in overestimation of intervention effectiveness. Our ongoing cluster randomised controlled QASC Australasia Trial will explore the relationship between facilitation dose and intervention outcomes [27]. Furthermore, facilitation can be costly with estimates of organisational facilitation costs (salary support for internal and external facilitators, facilitation support staff and stakeholders) from one study with four clinics said to be as high as US $263 490 during a 28-month period [67]. Further, the higher the facilitation dose, the greater the costs [68]. Given the implications for resourcing of higher facilitation doses and content, precise reporting and measurement is warranted to ensure adequate use of limited resources. An economic evaluation is planned as part of the QASC Australasia Trial to estimate the costs of the low and high dose facilitation interventions and identify an effective and affordable facilitation model for the implementation of evidence-based stroke protocols. It is hoped that the findings from this trial will generate new knowledge on the impact of facilitation dose on intervention effectiveness and efficiency and contribute to the field of implementation science.

As our scoping review evaluated facilitation as an implementation science strategy to improve the uptake of stroke and/or TIA interventions, we compared our findings to the newly updated Cochrane review by Lynch et al. [69] which evaluated the effects of implementation interventions in improving the delivery of evidence‐based stroke care. Of the seven acute stroke improvement intervention RCTs included in the review, six involved facilitators (referred to as change agents, site champions, quality improvement advisors or quality coordinator) in delivering or supporting the implementation of the intervention [25, 70–74]. The six RCTs only reported on the frequency and/or duration of different facilitation activities – a single 2.5 h interactive education session and workshop [73]; two face-to-face workshops and one site meeting [70]; two 30 to 60 min education sessions, one 60 min barrier identification and strategy development workshop and monthly phone or email contact for four months [71]; weekly online sharing and learning sessions [72]; two one-hour face-to-face multidisciplinary team workshops, and a 30-min education session [25]. The roles of facilitators were broad and involved activities such as leading education and working groups, goal setting, team building, performance feedback and action planning. Four RCTs [70–73] reported using guidelines or checklists, but only two [71, 72] provided completed checklists as supplementary material. These findings are consistent with our scoping review and emphasise an important gap in the implementation science literature regarding measurement and reporting on specific details of implementation strategies.

Overall, our findings are comparable to the non-stroke literature. Facilitation dose was also often measured based on frequency and/or duration (time) of facilitation Garner etl al. [74] tested an implementation and sustainment facilitation strategy for helping HIV organisations implement an intervention to decrease substance use disorders in their clients. Facilitation dose was measured in hours to reflect the frequency and duration of facilitation (maximum possible dose of 30 h comprising 18 h for up to 18 monthly virtual external facilitation meetings lasting up to 1 h each; and 12 h for up to two in-person facilitation meetings lasting up to 6 h each). Bucknall et al. [75] investigated the effectiveness of a facilitation intervention to improve nurses’ response to patient deterioration. Dose was measured as time allocated for facilitation, for example the internal hospital facilitator provided 5 h of support per week to intervention wards for 6 months [75]. In the systematic review by Baskerville et al. [76] which evaluated practice facilitation for the implementation of evidence-based practice guidelines within primary care practice settings, the authors measured facilitation intensity or dose by multiplying the mean number of contacts with a practice by the mean meeting time in hours. Sarkies et al. [77] evaluated the effectiveness of a knowledge broker strategy to facilitate evidence-informed resource allocation to inpatient weekend allied health services. Facilitation dose was described as the frequency of contacts over a 12-month period [77]. Similarly, the facilitation activities and facilitator roles in non-stroke studies were broad – readiness assessment, barrier and facilitator identification, ongoing training and consultation [78]; local needs assessment and plan development [77]; development of quality improvement tools, readiness assessment and barrier identification, identification and preparation of champions [79]; action plan development, auditing, care plan development and staff support to complete assessment forms [21].

Our findings, in addition to those from the non-stroke literature, have implications for the wider field of implementation science with the potential to contribute to the body of knowledge on facilitation as an implementation strategy. Without understanding facilitation strategies used, we cannot truly understand facilitation effectiveness. Consequently, this limits our ability to develop effective facilitation processes to maximise research use, guide facilitator behaviours, and determine the appropriate dose and content of facilitation [17]. Given the lack of a consistent measurement approach across stroke and other disciplines, we recommend that implementation science researchers should consider the development and validation of standardised methods (quantitative and qualitative) for measuring facilitation dose. In addition, the breadth of facilitation activities and roles of facilitators highlight the need for future studies to better operationalise the definition of facilitation by examining what elements of the role and activities could be delivered in fixed and discretionary ways. We also suggest embedding process evaluations into intervention effectiveness studies which use facilitation either as a discrete or part of a multifaceted implementation strategy to have a better understanding of the impact of context on facilitation. Encouraging use of standardised reporting guidelines for implementation studies may also help promote the explicit reporting of facilitation dose and content and improve transparency and rigor in research [80–85].

Our scoping review is limited by the inclusion of studies which evaluated interventions in patients with stroke and/or TIA in acute and/or subacute care settings. Therefore, our findings may lack generalisability beyond these settings. While measurement of facilitation dose has been attempted in general practice [76] and long-term care [21] settings, the focus of this review was on stroke implementation interventions which are typically provided in acute and subacute care (in-patient) settings. Given the wide variation in terminology used to describe different implementation support roles [86], we may have missed studies that used facilitation as an implementation strategy despite including common terms for this concept in our search strategy. Despite these limitations, our scoping review contributes to the body of knowledge on facilitation as an implementation strategy for evidence translation and sheds light on a critical aspect of implementation science – dose.

## Conclusion

This scoping review examined the evidence regarding the concepts of facilitation dose and content for implementing evidence-based stroke interventions. The findings of this review have the potential to better operationalise the measurement and reporting of facilitation. Further research on the impact of facilitation dose and content on intervention effectiveness and efficiency is needed to advance the field of implementation science.

## Supplementary Information

Additional file 1. Preferred Reporting Items for Systematic reviews and Meta-Analyses extension for Scoping Reviews (PRISMA-ScR) Checklist

Additional file 2. Search strategy

## Data Availability

Not applicable.

## Abbreviations

CONSORT: Consolidated Standards of Reporting Trials
QASC: Quality in Acute Stroke Care
RCTs: Randomised controlled trials
SPIRIT: Standard Protocol Items: Recommendations for Interventional Trials
SQUIRE: Standards for QUality Improvement Reporting Excellence
StaRI: Standards for Reporting Implementation Studies
TIA: Transient ischemic attack
TIDier: Template for Intervention Description and Replication
