# Supplementary material for: Finding the right dose: a scoping review examining facilitation as an implementation strategy for evidence-based stroke care
Source: Implement Sci. 2025 Jan 13;20:4. doi: 10.1186/s13012-025-01415-w (PMC11731140; doi:10.1186/s13012-025-01415-w)
Supplement: Supplementary file 2 — Additional file 2. Search strategy [file 13012_2025_1415_MOESM2_ESM.docx]

**Additional file 2: Search strategy**

Manuscript title: Finding the right dose: A scoping review examining facilitation as an implementation strategy for evidence-based stroke care.

Authors: Oyebola Fasugba, Heilok Cheng, Simeon Dale, Kelly Coughlan, Elizabeth McInnes, Dominique A Cadilhac, Ngai W Cheung, Kelvin Hill, Kirsty Page, Estela Sanjuan Menendez, Emily Neal, Vivien Pollnow, Julia Slark, Eileen Gilder, Anna Ranta, Christopher Levi, Jeremy M Grimshaw, Sandy Middleton

# Updated CINAHL search, done 11 July 2023

| **Search Terms** | **Search Options** | **Actions** | |  |  |
| --- | --- | --- | --- | --- | --- |
| S25 | S11 AND S18 AND S24 | | **Limiters** - Published Date: 20220301-20230731  **Expanders** - Apply equivalent subjects  **Search modes** - Boolean/Phrase | | [**View Results**](javascript:__doPostBack('ctl00$ctl00$FindField$FindField$historyControl$HistoryRepeater$ctl00$linkResults','')) (1,032)  [**View Details**](javascript:showShDetails(%22ctl00_ctl00_FindField_FindField_historyControl_ctrlPopup%22,%20%22S25%22,%20true);)  [**Edit**](https://web-s-ebscohost-com.ezproxy.library.sydney.edu.au/Legacy/Views/UserControls/Ehost/) |
| S24 | S19 OR S20 OR S21 OR S22 OR S23 | | **Expanders** - Apply equivalent subjects  **Search modes** - Boolean/Phrase | | [**View Results**](javascript:__doPostBack('ctl00$ctl00$FindField$FindField$historyControl$HistoryRepeater$ctl01$linkResults','')) (2,599,200)  [**View Details**](javascript:showShDetails(%22ctl00_ctl00_FindField_FindField_historyControl_ctrlPopup%22,%20%22S24%22,%20true);)  [**Edit**](https://web-s-ebscohost-com.ezproxy.library.sydney.edu.au/Legacy/Views/UserControls/Ehost/) |
| S23 | TI "experiment" OR AB "experiment" | | **Expanders** - Apply equivalent subjects  **Search modes** - Boolean/Phrase | | [**View Results**](javascript:__doPostBack('ctl00$ctl00$FindField$FindField$historyControl$HistoryRepeater$ctl02$linkResults','')) (31,173)  [**View Details**](javascript:showShDetails(%22ctl00_ctl00_FindField_FindField_historyControl_ctrlPopup%22,%20%22S23%22,%20true);)  [**Edit**](https://web-s-ebscohost-com.ezproxy.library.sydney.edu.au/Legacy/Views/UserControls/Ehost/) |
| S22 | TI "study" OR AB "study" | | **Expanders** - Apply equivalent subjects  **Search modes** - Boolean/Phrase | | [**View Results**](javascript:__doPostBack('ctl00$ctl00$FindField$FindField$historyControl$HistoryRepeater$ctl03$linkResults','')) (2,224,477)  [**View Details**](javascript:showShDetails(%22ctl00_ctl00_FindField_FindField_historyControl_ctrlPopup%22,%20%22S22%22,%20true);)  [**Edit**](https://web-s-ebscohost-com.ezproxy.library.sydney.edu.au/Legacy/Views/UserControls/Ehost/) |
| S21 | TI "program" OR AB "program" | | **Expanders** - Apply equivalent subjects  **Search modes** - Boolean/Phrase | | [**View Results**](javascript:__doPostBack('ctl00$ctl00$FindField$FindField$historyControl$HistoryRepeater$ctl04$linkResults','')) (234,651)  [**View Details**](javascript:showShDetails(%22ctl00_ctl00_FindField_FindField_historyControl_ctrlPopup%22,%20%22S21%22,%20true);)  [**Edit**](https://web-s-ebscohost-com.ezproxy.library.sydney.edu.au/Legacy/Views/UserControls/Ehost/) |
| S20 | TI "trial" OR AB "trial" | | **Expanders** - Apply equivalent subjects  **Search modes** - Boolean/Phrase | | [**View Results**](javascript:__doPostBack('ctl00$ctl00$FindField$FindField$historyControl$HistoryRepeater$ctl05$linkResults','')) (281,387)  [**View Details**](javascript:showShDetails(%22ctl00_ctl00_FindField_FindField_historyControl_ctrlPopup%22,%20%22S20%22,%20true);)  [**Edit**](https://web-s-ebscohost-com.ezproxy.library.sydney.edu.au/Legacy/Views/UserControls/Ehost/) |
| S19 | TI "intervention" OR AB "intervention" | | **Expanders** - Apply equivalent subjects  **Search modes** - Boolean/Phrase | | [**View Results**](javascript:__doPostBack('ctl00$ctl00$FindField$FindField$historyControl$HistoryRepeater$ctl06$linkResults','')) (330,142)  [**View Details**](javascript:showShDetails(%22ctl00_ctl00_FindField_FindField_historyControl_ctrlPopup%22,%20%22S19%22,%20true);)  [**Edit**](https://web-s-ebscohost-com.ezproxy.library.sydney.edu.au/Legacy/Views/UserControls/Ehost/) |
| S18 | S12 OR S13 OR S14 OR S15 OR S16 OR S17 | | **Expanders** - Apply equivalent subjects  **Search modes** - Boolean/Phrase | | [**View Results**](javascript:__doPostBack('ctl00$ctl00$FindField$FindField$historyControl$HistoryRepeater$ctl07$linkResults','')) (162,859)  [**View Details**](javascript:showShDetails(%22ctl00_ctl00_FindField_FindField_historyControl_ctrlPopup%22,%20%22S18%22,%20true);)  [**Edit**](https://web-s-ebscohost-com.ezproxy.library.sydney.edu.au/Legacy/Views/UserControls/Ehost/) |
| S17 | TI "quality improv*" OR AB "quality improv*" | | **Expanders** - Apply equivalent subjects  **Search modes** - Boolean/Phrase | | [**View Results**](javascript:__doPostBack('ctl00$ctl00$FindField$FindField$historyControl$HistoryRepeater$ctl08$linkResults','')) (28,948)  [**View Details**](javascript:showShDetails(%22ctl00_ctl00_FindField_FindField_historyControl_ctrlPopup%22,%20%22S17%22,%20true);)  [**Edit**](https://web-s-ebscohost-com.ezproxy.library.sydney.edu.au/Legacy/Views/UserControls/Ehost/) |
| S16 | TI "implementation" OR AB "implementation" | | **Expanders** - Apply equivalent subjects  **Search modes** - Boolean/Phrase | | [**View Results**](javascript:__doPostBack('ctl00$ctl00$FindField$FindField$historyControl$HistoryRepeater$ctl09$linkResults','')) (137,759)  [**View Details**](javascript:showShDetails(%22ctl00_ctl00_FindField_FindField_historyControl_ctrlPopup%22,%20%22S16%22,%20true);)  [**Edit**](https://web-s-ebscohost-com.ezproxy.library.sydney.edu.au/Legacy/Views/UserControls/Ehost/) |
| S15 | ""dissemination science"" | | **Expanders** - Apply equivalent subjects  **Search modes** - Boolean/Phrase | | [**View Results**](javascript:__doPostBack('ctl00$ctl00$FindField$FindField$historyControl$HistoryRepeater$ctl10$linkResults','')) (217)  [**View Details**](javascript:showShDetails(%22ctl00_ctl00_FindField_FindField_historyControl_ctrlPopup%22,%20%22S15%22,%20true);)  [**Edit**](https://web-s-ebscohost-com.ezproxy.library.sydney.edu.au/Legacy/Views/UserControls/Ehost/) |
| S14 | TI "knowledge translation" OR AB "knowledge translation" | | **Expanders** - Apply equivalent subjects  **Search modes** - Boolean/Phrase | | [**View Results**](javascript:__doPostBack('ctl00$ctl00$FindField$FindField$historyControl$HistoryRepeater$ctl11$linkResults','')) (2,428)  [**View Details**](javascript:showShDetails(%22ctl00_ctl00_FindField_FindField_historyControl_ctrlPopup%22,%20%22S14%22,%20true);)  [**Edit**](https://web-s-ebscohost-com.ezproxy.library.sydney.edu.au/Legacy/Views/UserControls/Ehost/) |
| S13 | ""implementation science"" | | **Expanders** - Apply equivalent subjects  **Search modes** - Boolean/Phrase | | [**View Results**](javascript:__doPostBack('ctl00$ctl00$FindField$FindField$historyControl$HistoryRepeater$ctl12$linkResults','')) (2,750)  [**View Details**](javascript:showShDetails(%22ctl00_ctl00_FindField_FindField_historyControl_ctrlPopup%22,%20%22S13%22,%20true);)  [**Edit**](https://web-s-ebscohost-com.ezproxy.library.sydney.edu.au/Legacy/Views/UserControls/Ehost/) |
| S12 | (MH "Implementation Science") | | **Expanders** - Apply equivalent subjects  **Search modes** - Boolean/Phrase | | [**View Results**](javascript:__doPostBack('ctl00$ctl00$FindField$FindField$historyControl$HistoryRepeater$ctl13$linkResults','')) (971)  [**View Details**](javascript:showShDetails(%22ctl00_ctl00_FindField_FindField_historyControl_ctrlPopup%22,%20%22S12%22,%20true);)  [**Edit**](https://web-s-ebscohost-com.ezproxy.library.sydney.edu.au/Legacy/Views/UserControls/Ehost/) |
| S11 | S1 OR S2 OR S3 OR S4 OR S5 OR S6 OR S7 OR S8 OR S9 OR S10 | | **Expanders** - Apply equivalent subjects  **Search modes** - Boolean/Phrase | | [**View Results**](javascript:__doPostBack('ctl00$ctl00$FindField$FindField$historyControl$HistoryRepeater$ctl14$linkResults','')) (110,664)  [**View Details**](javascript:showShDetails(%22ctl00_ctl00_FindField_FindField_historyControl_ctrlPopup%22,%20%22S11%22,%20true);)  [**Edit**](https://web-s-ebscohost-com.ezproxy.library.sydney.edu.au/Legacy/Views/UserControls/Ehost/) |
| S10 | TI "educator" OR AB "educator" | | **Expanders** - Apply equivalent subjects  **Search modes** - Boolean/Phrase | | [**View Results**](javascript:__doPostBack('ctl00$ctl00$FindField$FindField$historyControl$HistoryRepeater$ctl15$linkResults','')) (6,624)  [**View Details**](javascript:showShDetails(%22ctl00_ctl00_FindField_FindField_historyControl_ctrlPopup%22,%20%22S10%22,%20true);)  [**Edit**](https://web-s-ebscohost-com.ezproxy.library.sydney.edu.au/Legacy/Views/UserControls/Ehost/) |
| S9 | TI "trainer" OR AB "trainer" | | **Expanders** - Apply equivalent subjects  **Search modes** - Boolean/Phrase | | [**View Results**](javascript:__doPostBack('ctl00$ctl00$FindField$FindField$historyControl$HistoryRepeater$ctl16$linkResults','')) (3,875)  [**View Details**](javascript:showShDetails(%22ctl00_ctl00_FindField_FindField_historyControl_ctrlPopup%22,%20%22S9%22,%20true);)  [**Edit**](https://web-s-ebscohost-com.ezproxy.library.sydney.edu.au/Legacy/Views/UserControls/Ehost/) |
| S8 | TI "mentor" OR AB "mentor" | | **Expanders** - Apply equivalent subjects  **Search modes** - Boolean/Phrase | | [**View Results**](javascript:__doPostBack('ctl00$ctl00$FindField$FindField$historyControl$HistoryRepeater$ctl17$linkResults','')) (4,427)  [**View Details**](javascript:showShDetails(%22ctl00_ctl00_FindField_FindField_historyControl_ctrlPopup%22,%20%22S8%22,%20true);)  [**Edit**](https://web-s-ebscohost-com.ezproxy.library.sydney.edu.au/Legacy/Views/UserControls/Ehost/) |
| S7 | TI "consultant" OR AB "consultant" | | **Expanders** - Apply equivalent subjects  **Search modes** - Boolean/Phrase | | [**View Results**](javascript:__doPostBack('ctl00$ctl00$FindField$FindField$historyControl$HistoryRepeater$ctl18$linkResults','')) (9,230)  [**View Details**](javascript:showShDetails(%22ctl00_ctl00_FindField_FindField_historyControl_ctrlPopup%22,%20%22S7%22,%20true);)  [**Edit**](https://web-s-ebscohost-com.ezproxy.library.sydney.edu.au/Legacy/Views/UserControls/Ehost/) |
| S6 | TI "coach" OR AB "coach" | | **Expanders** - Apply equivalent subjects  **Search modes** - Boolean/Phrase | | [**View Results**](javascript:__doPostBack('ctl00$ctl00$FindField$FindField$historyControl$HistoryRepeater$ctl19$linkResults','')) (3,699)  [**View Details**](javascript:showShDetails(%22ctl00_ctl00_FindField_FindField_historyControl_ctrlPopup%22,%20%22S6%22,%20true);)  [**Edit**](https://web-s-ebscohost-com.ezproxy.library.sydney.edu.au/Legacy/Views/UserControls/Ehost/) |
| S5 | ( TI "implement*" OR AB "implement*" ) AND ( TI "support" OR AB "support" ) AND ( TI "practitioner" OR AB "practitioner" ) | | **Expanders** - Apply equivalent subjects  **Search modes** - Boolean/Phrase | | [**View Results**](javascript:__doPostBack('ctl00$ctl00$FindField$FindField$historyControl$HistoryRepeater$ctl20$linkResults','')) (815)  [**View Details**](javascript:showShDetails(%22ctl00_ctl00_FindField_FindField_historyControl_ctrlPopup%22,%20%22S5%22,%20true);)  [**Edit**](https://web-s-ebscohost-com.ezproxy.library.sydney.edu.au/Legacy/Views/UserControls/Ehost/) |
| S4 | ""knowledge broker"" | | **Expanders** - Apply equivalent subjects  **Search modes** - Boolean/Phrase | | [**View Results**](javascript:__doPostBack('ctl00$ctl00$FindField$FindField$historyControl$HistoryRepeater$ctl21$linkResults','')) (204)  [**View Details**](javascript:showShDetails(%22ctl00_ctl00_FindField_FindField_historyControl_ctrlPopup%22,%20%22S4%22,%20true);)  [**Edit**](https://web-s-ebscohost-com.ezproxy.library.sydney.edu.au/Legacy/Views/UserControls/Ehost/) |
| S3 | TI "facilitate" OR AB "facilitate" | | **Expanders** - Apply equivalent subjects  **Search modes** - Boolean/Phrase | | [**View Results**](javascript:__doPostBack('ctl00$ctl00$FindField$FindField$historyControl$HistoryRepeater$ctl22$linkResults','')) (73,875)  [**View Details**](javascript:showShDetails(%22ctl00_ctl00_FindField_FindField_historyControl_ctrlPopup%22,%20%22S3%22,%20true);)  [**Edit**](https://web-s-ebscohost-com.ezproxy.library.sydney.edu.au/Legacy/Views/UserControls/Ehost/) |
| S2 | TI "facilitation" OR AB "facilitation" | | **Expanders** - Apply equivalent subjects  **Search modes** - Boolean/Phrase | | [**View Results**](javascript:__doPostBack('ctl00$ctl00$FindField$FindField$historyControl$HistoryRepeater$ctl23$linkResults','')) (7,243)  [**View Details**](javascript:showShDetails(%22ctl00_ctl00_FindField_FindField_historyControl_ctrlPopup%22,%20%22S2%22,%20true);)  [**Edit**](https://web-s-ebscohost-com.ezproxy.library.sydney.edu.au/Legacy/Views/UserControls/Ehost/) |
| S1 | TI "facilitator" OR AB "facilitator" | | **Expanders** - Apply equivalent subjects  **Search modes** - Boolean/Phrase | | [**View Results**](javascript:__doPostBack('ctl00$ctl00$FindField$FindField$historyControl$HistoryRepeater$ctl24$linkResults','')) (3,952)  [**View Details**](javascript:showShDetails(%22ctl00_ctl00_FindField_FindField_historyControl_ctrlPopup%22,%20%22S1%22,%20true);)  [**Edit**](https://web-s-ebscohost-com.ezproxy.library.sydney.edu.au/Legacy/Views/UserControls/Ehost/) |

# Initial CINAHL search, done 19 March 2022

| **Search Terms** | **Search Options** | **Actions** |  |
| --- | --- | --- | --- |
| 26 | S11 AND S18 AND S24 | **Expanders** - Apply equivalent subjects  **Search modes** - Boolean/Phrase | [**View Results**](javascript:__doPostBack('ctl00$ctl00$FindField$FindField$historyControl$HistoryRepeater$ctl00$linkResults','')) (3,753)  [**View Details**](javascript:showShDetails(%22ctl00_ctl00_FindField_FindField_historyControl_ctrlPopup%22,%20%22S25%22);)  [**Edit**](https://web-s-ebscohost-com.ezproxy.library.sydney.edu.au/Legacy/Views/UserControls/Ehost/) |
| S25 | S11 AND S18 AND S24 | **Expanders** - Apply equivalent subjects  **Search modes** - Boolean/Phrase | [**View Results**](javascript:__doPostBack('ctl00$ctl00$FindField$FindField$historyControl$HistoryRepeater$ctl00$linkResults','')) (7,030)  [**View Details**](javascript:showShDetails(%22ctl00_ctl00_FindField_FindField_historyControl_ctrlPopup%22,%20%22S25%22);)  [**Edit**](https://web-s-ebscohost-com.ezproxy.library.sydney.edu.au/Legacy/Views/UserControls/Ehost/) |
| S24 | S19 OR S20 OR S21 OR S22 OR S23 | **Expanders** - Apply equivalent subjects  **Search modes** - Boolean/Phrase | [**View Results**](javascript:__doPostBack('ctl00$ctl00$FindField$FindField$historyControl$HistoryRepeater$ctl01$linkResults','')) (2,383,731)  [**View Details**](javascript:showShDetails(%22ctl00_ctl00_FindField_FindField_historyControl_ctrlPopup%22,%20%22S24%22);)  [**Edit**](https://web-s-ebscohost-com.ezproxy.library.sydney.edu.au/Legacy/Views/UserControls/Ehost/) |
| S23 | TI "experiment" OR AB "experiment" | **Expanders** - Apply equivalent subjects  **Search modes** - Boolean/Phrase | [**View Results**](javascript:__doPostBack('ctl00$ctl00$FindField$FindField$historyControl$HistoryRepeater$ctl02$linkResults','')) (28,834)  [**View Details**](javascript:showShDetails(%22ctl00_ctl00_FindField_FindField_historyControl_ctrlPopup%22,%20%22S23%22);)  [**Edit**](https://web-s-ebscohost-com.ezproxy.library.sydney.edu.au/Legacy/Views/UserControls/Ehost/) |
| S22 | TI "study" OR AB "study" | **Expanders** - Apply equivalent subjects  **Search modes** - Boolean/Phrase | [**View Results**](javascript:__doPostBack('ctl00$ctl00$FindField$FindField$historyControl$HistoryRepeater$ctl03$linkResults','')) (2,033,722)  [**View Details**](javascript:showShDetails(%22ctl00_ctl00_FindField_FindField_historyControl_ctrlPopup%22,%20%22S22%22);)  [**Edit**](https://web-s-ebscohost-com.ezproxy.library.sydney.edu.au/Legacy/Views/UserControls/Ehost/) |
| S21 | TI "program" OR AB "program" | **Expanders** - Apply equivalent subjects  **Search modes** - Boolean/Phrase | [**View Results**](javascript:__doPostBack('ctl00$ctl00$FindField$FindField$historyControl$HistoryRepeater$ctl04$linkResults','')) (221,138)  [**View Details**](javascript:showShDetails(%22ctl00_ctl00_FindField_FindField_historyControl_ctrlPopup%22,%20%22S21%22);)  [**Edit**](https://web-s-ebscohost-com.ezproxy.library.sydney.edu.au/Legacy/Views/UserControls/Ehost/) |
| S20 | TI "trial" OR AB "trial" | **Expanders** - Apply equivalent subjects  **Search modes** - Boolean/Phrase | [**View Results**](javascript:__doPostBack('ctl00$ctl00$FindField$FindField$historyControl$HistoryRepeater$ctl05$linkResults','')) (256,681)  [**View Details**](javascript:showShDetails(%22ctl00_ctl00_FindField_FindField_historyControl_ctrlPopup%22,%20%22S20%22);)  [**Edit**](https://web-s-ebscohost-com.ezproxy.library.sydney.edu.au/Legacy/Views/UserControls/Ehost/) |
| S19 | TI "intervention" OR AB "intervention" | **Expanders** - Apply equivalent subjects  **Search modes** - Boolean/Phrase | [**View Results**](javascript:__doPostBack('ctl00$ctl00$FindField$FindField$historyControl$HistoryRepeater$ctl06$linkResults','')) (300,922)  [**View Details**](javascript:showShDetails(%22ctl00_ctl00_FindField_FindField_historyControl_ctrlPopup%22,%20%22S19%22);)  [**Edit**](https://web-s-ebscohost-com.ezproxy.library.sydney.edu.au/Legacy/Views/UserControls/Ehost/) |
| S18 | S12 OR S13 OR S14 OR S15 OR S16 OR S17 | **Expanders** - Apply equivalent subjects  **Search modes** - Boolean/Phrase | [**View Results**](javascript:__doPostBack('ctl00$ctl00$FindField$FindField$historyControl$HistoryRepeater$ctl07$linkResults','')) (147,006)  [**View Details**](javascript:showShDetails(%22ctl00_ctl00_FindField_FindField_historyControl_ctrlPopup%22,%20%22S18%22);)  [**Edit**](https://web-s-ebscohost-com.ezproxy.library.sydney.edu.au/Legacy/Views/UserControls/Ehost/) |
| S17 | TI "quality improv*" OR AB "quality improv*" | **Expanders** - Apply equivalent subjects  **Search modes** - Boolean/Phrase | [**View Results**](javascript:__doPostBack('ctl00$ctl00$FindField$FindField$historyControl$HistoryRepeater$ctl08$linkResults','')) (26,286)  [**View Details**](javascript:showShDetails(%22ctl00_ctl00_FindField_FindField_historyControl_ctrlPopup%22,%20%22S17%22);)  [**Edit**](https://web-s-ebscohost-com.ezproxy.library.sydney.edu.au/Legacy/Views/UserControls/Ehost/) |
| S16 | TI "implementation" OR AB "implementation" | **Expanders** - Apply equivalent subjects  **Search modes** - Boolean/Phrase | [**View Results**](javascript:__doPostBack('ctl00$ctl00$FindField$FindField$historyControl$HistoryRepeater$ctl09$linkResults','')) (124,224)  [**View Details**](javascript:showShDetails(%22ctl00_ctl00_FindField_FindField_historyControl_ctrlPopup%22,%20%22S16%22);)  [**Edit**](https://web-s-ebscohost-com.ezproxy.library.sydney.edu.au/Legacy/Views/UserControls/Ehost/) |
| S15 | "dissemination science" | **Expanders** - Apply equivalent subjects  **Search modes** - Boolean/Phrase | [**View Results**](javascript:__doPostBack('ctl00$ctl00$FindField$FindField$historyControl$HistoryRepeater$ctl10$linkResults','')) (10)  [**View Details**](javascript:showShDetails(%22ctl00_ctl00_FindField_FindField_historyControl_ctrlPopup%22,%20%22S15%22);)  [**Edit**](https://web-s-ebscohost-com.ezproxy.library.sydney.edu.au/Legacy/Views/UserControls/Ehost/) |
| S14 | TI "knowledge translation" OR AB "knowledge translation" | **Expanders** - Apply equivalent subjects  **Search modes** - Boolean/Phrase | [**View Results**](javascript:__doPostBack('ctl00$ctl00$FindField$FindField$historyControl$HistoryRepeater$ctl11$linkResults','')) (2,222)  [**View Details**](javascript:showShDetails(%22ctl00_ctl00_FindField_FindField_historyControl_ctrlPopup%22,%20%22S14%22);)  [**Edit**](https://web-s-ebscohost-com.ezproxy.library.sydney.edu.au/Legacy/Views/UserControls/Ehost/) |
| S13 | "implementation science" | **Expanders** - Apply equivalent subjects  **Search modes** - Boolean/Phrase | [**View Results**](javascript:__doPostBack('ctl00$ctl00$FindField$FindField$historyControl$HistoryRepeater$ctl12$linkResults','')) (1,758)  [**View Details**](javascript:showShDetails(%22ctl00_ctl00_FindField_FindField_historyControl_ctrlPopup%22,%20%22S13%22);)  [**Edit**](https://web-s-ebscohost-com.ezproxy.library.sydney.edu.au/Legacy/Views/UserControls/Ehost/) |
| S12 | (MH "Implementation Science") | **Expanders** - Apply equivalent subjects  **Search modes** - Boolean/Phrase | [**View Results**](javascript:__doPostBack('ctl00$ctl00$FindField$FindField$historyControl$HistoryRepeater$ctl13$linkResults','')) (512)  [**View Details**](javascript:showShDetails(%22ctl00_ctl00_FindField_FindField_historyControl_ctrlPopup%22,%20%22S12%22);)  [**Edit**](https://web-s-ebscohost-com.ezproxy.library.sydney.edu.au/Legacy/Views/UserControls/Ehost/) |
| S11 | S1 OR S2 OR S3 OR S4 OR S5 OR S6 OR S7 OR S8 OR S9 OR S10 | **Expanders** - Apply equivalent subjects  **Search modes** - Boolean/Phrase | [**View Results**](javascript:__doPostBack('ctl00$ctl00$FindField$FindField$historyControl$HistoryRepeater$ctl14$linkResults','')) (102,101)  [**View Details**](javascript:showShDetails(%22ctl00_ctl00_FindField_FindField_historyControl_ctrlPopup%22,%20%22S11%22);)  [**Edit**](https://web-s-ebscohost-com.ezproxy.library.sydney.edu.au/Legacy/Views/UserControls/Ehost/) |
| S10 | TI "educator" OR AB "educator" | **Expanders** - Apply equivalent subjects  **Search modes** - Boolean/Phrase | [**View Results**](javascript:__doPostBack('ctl00$ctl00$FindField$FindField$historyControl$HistoryRepeater$ctl15$linkResults','')) (6,338)  [**View Details**](javascript:showShDetails(%22ctl00_ctl00_FindField_FindField_historyControl_ctrlPopup%22,%20%22S10%22);)  [**Edit**](https://web-s-ebscohost-com.ezproxy.library.sydney.edu.au/Legacy/Views/UserControls/Ehost/) |
| S9 | TI "trainer" OR AB "trainer" | **Expanders** - Apply equivalent subjects  **Search modes** - Boolean/Phrase | [**View Results**](javascript:__doPostBack('ctl00$ctl00$FindField$FindField$historyControl$HistoryRepeater$ctl16$linkResults','')) (3,733)  [**View Details**](javascript:showShDetails(%22ctl00_ctl00_FindField_FindField_historyControl_ctrlPopup%22,%20%22S9%22);)  [**Edit**](https://web-s-ebscohost-com.ezproxy.library.sydney.edu.au/Legacy/Views/UserControls/Ehost/) |
| S8 | TI "mentor" OR AB "mentor" | **Expanders** - Apply equivalent subjects  **Search modes** - Boolean/Phrase | [**View Results**](javascript:__doPostBack('ctl00$ctl00$FindField$FindField$historyControl$HistoryRepeater$ctl17$linkResults','')) (4,196)  [**View Details**](javascript:showShDetails(%22ctl00_ctl00_FindField_FindField_historyControl_ctrlPopup%22,%20%22S8%22);)  [**Edit**](https://web-s-ebscohost-com.ezproxy.library.sydney.edu.au/Legacy/Views/UserControls/Ehost/) |
| S7 | TI "consultant" OR AB "consultant" | **Expanders** - Apply equivalent subjects  **Search modes** - Boolean/Phrase | [**View Results**](javascript:__doPostBack('ctl00$ctl00$FindField$FindField$historyControl$HistoryRepeater$ctl18$linkResults','')) (9,024)  [**View Details**](javascript:showShDetails(%22ctl00_ctl00_FindField_FindField_historyControl_ctrlPopup%22,%20%22S7%22);)  [**Edit**](https://web-s-ebscohost-com.ezproxy.library.sydney.edu.au/Legacy/Views/UserControls/Ehost/) |
| S6 | TI "coach" OR AB "coach" | **Expanders** - Apply equivalent subjects  **Search modes** - Boolean/Phrase | [**View Results**](javascript:__doPostBack('ctl00$ctl00$FindField$FindField$historyControl$HistoryRepeater$ctl19$linkResults','')) (3,405)  [**View Details**](javascript:showShDetails(%22ctl00_ctl00_FindField_FindField_historyControl_ctrlPopup%22,%20%22S6%22);)  [**Edit**](https://web-s-ebscohost-com.ezproxy.library.sydney.edu.au/Legacy/Views/UserControls/Ehost/) |
| S5 | ( TI "implement*" OR AB "implement*" ) AND ( TI "support" OR AB "support" ) AND ( TI "practitioner" OR AB "practitioner" ) | **Expanders** - Apply equivalent subjects  **Search modes** - Boolean/Phrase | [**View Results**](javascript:__doPostBack('ctl00$ctl00$FindField$FindField$historyControl$HistoryRepeater$ctl20$linkResults','')) (720)  [**View Details**](javascript:showShDetails(%22ctl00_ctl00_FindField_FindField_historyControl_ctrlPopup%22,%20%22S5%22);)  [**Edit**](https://web-s-ebscohost-com.ezproxy.library.sydney.edu.au/Legacy/Views/UserControls/Ehost/) |
| S4 | "knowledge broker" | **Expanders** - Apply equivalent subjects  **Search modes** - Boolean/Phrase | [**View Results**](javascript:__doPostBack('ctl00$ctl00$FindField$FindField$historyControl$HistoryRepeater$ctl21$linkResults','')) (63)  [**View Details**](javascript:showShDetails(%22ctl00_ctl00_FindField_FindField_historyControl_ctrlPopup%22,%20%22S4%22);)  [**Edit**](https://web-s-ebscohost-com.ezproxy.library.sydney.edu.au/Legacy/Views/UserControls/Ehost/) |
| S3 | TI "facilitate" OR AB "facilitate" | **Expanders** - Apply equivalent subjects  **Search modes** - Boolean/Phrase | [**View Results**](javascript:__doPostBack('ctl00$ctl00$FindField$FindField$historyControl$HistoryRepeater$ctl22$linkResults','')) (67,321)  [**View Details**](javascript:showShDetails(%22ctl00_ctl00_FindField_FindField_historyControl_ctrlPopup%22,%20%22S3%22);)  [**Edit**](https://web-s-ebscohost-com.ezproxy.library.sydney.edu.au/Legacy/Views/UserControls/Ehost/) |
| S2 | TI "facilitation" OR AB "facilitation" | **Expanders** - Apply equivalent subjects  **Search modes** - Boolean/Phrase | [**View Results**](javascript:__doPostBack('ctl00$ctl00$FindField$FindField$historyControl$HistoryRepeater$ctl23$linkResults','')) (6,729)  [**View Details**](javascript:showShDetails(%22ctl00_ctl00_FindField_FindField_historyControl_ctrlPopup%22,%20%22S2%22);)  [**Edit**](https://web-s-ebscohost-com.ezproxy.library.sydney.edu.au/Legacy/Views/UserControls/Ehost/) |
| S1 | TI "facilitator" OR AB "facilitator" | **Expanders** - Apply equivalent subjects  **Search modes** - Boolean/Phrase | [**View Results**](javascript:__doPostBack('ctl00$ctl00$FindField$FindField$historyControl$HistoryRepeater$ctl24$linkResults','')) (3,535)  [**View Details**](javascript:showShDetails(%22ctl00_ctl00_FindField_FindField_historyControl_ctrlPopup%22,%20%22S1%22);)  [**Edit**](https://web-s-ebscohost-com.ezproxy.library.sydney.edu.au/Legacy/Views/UserControls/Ehost/) |

# Updated Medline search, done 11 July 2023

| [**# ▲**](https://ovidsp-dc2-ovid-com.ezproxy.library.sydney.edu.au/ovid-new-a/ovidweb.cgi?&S=FDKAFPEANGEBDFMHJPLJNHIHIMKDAA00&Sort+Sets=descending) | **Searches** | **Results** |
| --- | --- | --- |
| 1 | facilitator.ti,ab,kw. | 8635 |
| 2 | facilitation.ti,ab,kw. | 33993 |
| 3 | facilitate.ti,ab,kw. | 343160 |
| 4 | "knowledge broker".ti,ab,kw. | 86 |
| 5 | implement*.ti,ab,kw. | 695311 |
| 6 | support.ti,ab,kw. | 1254663 |
| 7 | practitioner.ti,ab,kw. | 55791 |
| 8 | 5 and 6 and 7 | 1078 |
| 9 | coach.ti,ab,kw. | 4626 |
| 10 | consultant.ti,ab,kw. | 15570 |
| 11 | mentor.ti,ab,kw. | 6556 |
| 12 | trainer.ti,ab,kw. | 5747 |
| 13 | educator.ti,ab,kw. | 6811 |
| 14 | 1 or 2 or 3 or 4 or 8 or 9 or 10 or 11 or 12 or 13 | 419828 |
| 15 | Implementation Science/ | 1283 |
| 16 | "implementation science".mp. | 5898 |
| 17 | "knowledge translation".ti,ab,kw. | 4373 |
| 18 | "dissemination science".mp. | 35 |
| 19 | "quality improv*".ti,ab,kw. | 55047 |
| 20 | 15 or 16 or 17 or 18 or 19 | 64296 |
| 21 | intervention.ti,ab,kw. | 790711 |
| 22 | trial.ti,ab,kw. | 759990 |
| 23 | program.ti,ab,kw. | 529385 |
| 24 | study.ti,ab,kw. | 9740642 |
| 25 | experiment.ti,ab,kw. | 382651 |
| 26 | 21 or 22 or 23 or 24 or 25 | 10860775 |
| 27 | 14 and 20 and 26 | 3041 |
| 28 | limit 27 to yr="2022 -Current" | 652 |

# Initial Medline search, done 19 March 2022

| [# ▲](https://ovidsp-dc2-ovid-com.ezproxy.library.sydney.edu.au/ovid-b/ovidweb.cgi?&S=EOFFFPDNEHEBKMAJIPNJNGPFEIJOAA00&Sort+Sets=descending) | **Searches** | **Results** |
| --- | --- | --- |
| 1 | facilitator.ti,ab,kw. | 7542 |
| 2 | facilitation.ti,ab,kw. | 32038 |
| 3 | facilitate.ti,ab,kw. | 305225 |
| 4 | "knowledge broker".ti,ab,kw. | 83 |
| 5 | implement*.ti,ab,kw. | 601534 |
| 6 | support.ti,ab,kw. | 1131805 |
| 7 | practitioner.ti,ab,kw. | 52360 |
| 8 | 5 and 6 and 7 | 930 |
| 9 | coach.ti,ab,kw. | 3922 |
| 10 | consultant.ti,ab,kw. | 14756 |
| 11 | mentor.ti,ab,kw. | 5910 |
| 12 | trainer.ti,ab,kw. | 5232 |
| 13 | educator.ti,ab,kw. | 6180 |
| 14 | 1 or 2 or 3 or 4 or 8 or 9 or 10 or 11 or 12 or 13 | 376023 |
| 15 | Implementation Science/ | 1038 |
| 16 | "implementation science".mp. | 4410 |
| 17 | "knowledge translation".ti,ab,kw. | 3828 |
| 18 | "dissemination science".mp. | 35 |
| 19 | "quality improv*".ti,ab,kw. | 48150 |
| 20 | 15 or 16 or 17 or 18 or 19 | 55590 |
| 21 | intervention.ti,ab,kw. | 703845 |
| 22 | trial.ti,ab,kw. | 687663 |
| 23 | program.ti,ab,kw. | 488015 |
| 24 | study.ti,ab,kw. | 8811508 |
| 25 | experiment.ti,ab,kw. | 350982 |
| 26 | 21 or 22 or 23 or 24 or 25 | 9851594 |
| 27 | 14 and 20 and 26 | 350982 |
| 28 | limit 27 to yr="2017 - Current" | 1568 |

# Updated Cochrane search, done 18 July 2023

Search Name:

Date Run: 18/07/2023 05:24:50

Comment:

ID Search Hits

#1 ((facilitator):ti,ab,kw (Word variations have been searched)):ti,ab,kw (Word variations have been searched) 36800

#2 (facilitation):ti,ab,kw 3514

#3 (facilitate):ti,ab,kw 17492

#4 ("knowledge broker"):ti,ab,kw 15

#5 (implement*):ti,ab,kw and (support):ti,ab,kw and (practitioner):ti,ab,kw 490

#6 (coach):ti,ab,kw 2282

#7 (consultant):ti,ab,kw 2687

#8 (mentor):ti,ab,kw 909

#9 (trainer):ti,ab,kw 2333

#10 #1 or #2 or #3 #4 or #5 or #6 or #7 or #8 or #9 44548

#11 ("implementation science"):ti,ab,kw 780

#12 (implementation):ti,ab,kw 29617

#13 ("knowledge translation"):ti,ab,kw 376

#14 ("dissemination science"):ti,ab,kw 4

#15 ("qual* improvement"):ti,ab,kw 25

#16 #11 or #12 or #13 or #14 or #15 29879

#17 (intervention):ti,ab,kw 496695

#18 (trial):ti,ab,kw 1013903

#19 (program):ti,ab,kw 133058

#20 (study):ti,ab,kw 1379471

#21 (experiment):ti,ab,kw 63461

#22 #17 or #18 or #19 or #20 or #21 1696625

#23 #10 and #16 and #22 with Cochrane Library publication date Between Jan 2022 and Jul 2023 1193

NB: 1193 references include 1 protocol and 1 editorial, not downloaded

# Initial Cochrane search, done 19 March 2022

Search Name:

Date Run: 18/03/2022 21:03:28

Comment:

ID Search Hits

#1 (facilitator):ti,ab,kw (Word variations have been searched) 32046

#2 (facilitation):ti,ab,kw 3032

#3 (facilitate):ti,ab,kw 15336

#4 ("knowledge broker"):ti,ab,kw 15

#5 (implement*):ti,ab,kw and (support):ti,ab,kw and (practitioner):ti,ab,kw 399

#6 (coach):ti,ab,kw 1882

#7 (consultant):ti,ab,kw 2242

#8 (mentor):ti,ab,kw 786

#9 (trainer):ti,ab,kw 2036

#10 #1 or #2 or #3 #4 or #5 or #6 or #7 or #8 or #9 38609

#11 ("implementation science"):ti,ab,kw 577

#12 (implementation):ti,ab,kw 24850

#13 ("knowledge translation"):ti,ab,kw 350

#14 ("dissemination science"):ti,ab,kw 4

#15 ("qual* improvement"):ti,ab,kw 20

#16 #11 or #12 or #13 or #14 or #15 25097

#17 (intervention):ti,ab,kw 413356

#18 (trial):ti,ab,kw 921638

#19 (program):ti,ab,kw 118022

#20 (study):ti,ab,kw 1251795

#21 (experiment):ti,ab,kw 54969

#22 #17 or #18 or #19 or #20 or #21 1535784

#23 #10 and #16 and #22 3289

NB: 3289 references include 4 protocols and 13 editorials, not downloaded
